# Click chemistry-based drug delivery systems for targeted cancer therapy

**DOI:** 10.1016/j.mtbio.2026.102912

**Published:** 2026-02-12

**Authors:** Yonghui Liu, Wei Zhang, Yanan Wu, Dong Wan, Jie Pan

**Affiliations:** a School of Chemistry, Tiangong University, Tianjin, 300387, China; b School of Chemical Engineering and Technology, Tiangong University, Tianjin, 300387, China

**Keywords:** Click chemistry, Drug delivery systems, Actively targeted, Cancer treatment

## Abstract

Drug delivery systems (DDSs) play a crucial role in improving the efficacy and reducing the side effects of cancer treatment. However, traditional DDSs face challenges such as poor targeting, tissue penetration, and uncontrolled drug release. Click chemistry offers a powerful tool for addressing these limitations by enabling precise modification and targeting of DDSs. This review explores the application of click chemistry in the construction of active targeted DDSs for cancer therapy, focusing on two key strategies: in vitro and in vivo. In vitro strategies involve direct coupling of targeting agents to carrier materials, while in vivo strategies utilize metabolic engineering and click chemistry for cell labeling and drug delivery. The review discusses the advantages and limitations of different click chemistry reactions, including copper-catalyzed azide-alkyne cycloaddition (CuAAC), strain-promoted azide-alkyne cycloaddition (SPAAC), inverse electron-demand Diels-Alder (IEDDA) reaction, thiol-ene reaction, sulfur (VI) fluoride exchange (SuFEx) reaction, and selenium-nitrogen exchange (SeNEx) reaction. It also highlights recent advancements in using click chemistry to construct multifunctional DDSs, such as tumor-targeted biomimetic systems and cell-based delivery systems. Finally, the review outlines the challenges and future directions of click chemistry in drug delivery, emphasizing the need for precise control, expanded toolkits, and integration with emerging technologies to create intelligent, multifunctional DDSs with enhanced therapeutic efficacy and reduced side effects.

## Graphical abstract

**Figure:**
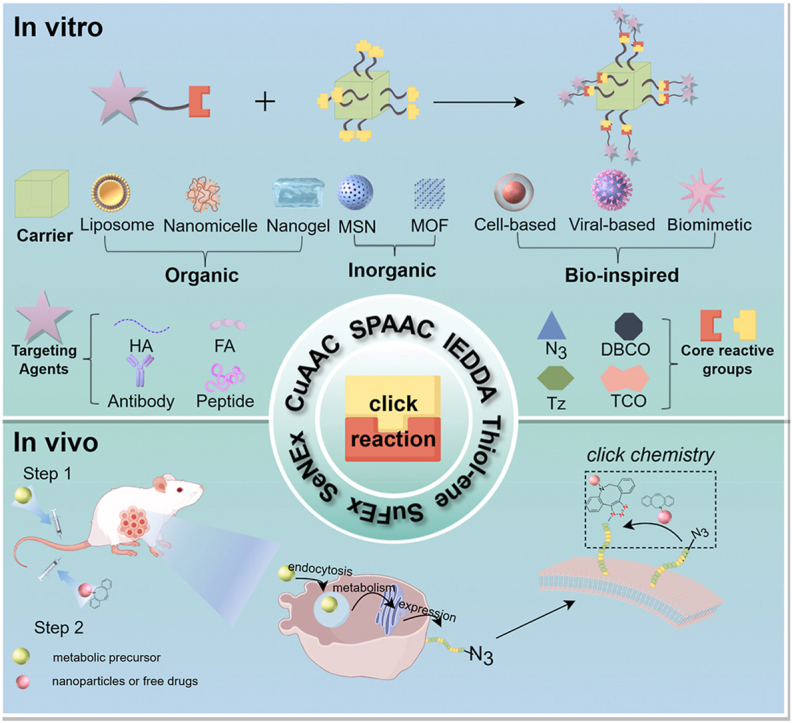

## 1 Introduction

Drug delivery systems (DDSs) are primarily aimed at precisely delivering therapeutic agents to diseased sites [1,2], thereby enhancing efficacy and reducing toxic side effects by improving target enrichment and minimizing off-target distribution [[3], [4], [5], [6]]. However, conventional delivery strategies have consistently been hindered by limitations such as insufficient targeting accuracy [7], poor tissue penetration, and uncontrolled drug release [8]. The fundamental process typically relies on carrier systems for drug loading and delivery [9]: synthetic organic carriers (e.g., liposomes [10], polymeric micelles [11,12]), inorganic carriers (e.g., metal-organic frameworks [13], mesoporous silica nanoparticles [14]), and bio-inspired carriers (e.g., cell-based [15], virus-based vectors [16]) are first loaded with therapeutic agents like small molecule chemotherapeutics, nucleic acids, and antibodies [17]. These carriers then reach target areas via passive or active targeting, ultimately releasing drugs in response to the microenvironment [[18], [19], [20]].

From a mechanistic perspective, passive targeting primarily relies on the enhanced permeability and retention (EPR) effect [21,22], where nanoscale carriers accumulate in pathological tissues such as tumors due to the high permeability of their vascular walls and impaired lymphatic drainage [23]. However, this approach has significant limitations. Carriers tend to aggregate in perivascular regions, struggling to penetrate dense tumor stroma or inflammatory tissue matrices, leading to insufficient drug concentration in deep lesions [24,25]. Active targeting, on the other hand, involves modifying the carrier surface with ligands such as folate [18], RGD peptides, or antibodies, enabling specific binding through receptors overexpressed on target cells [26,27]. Nevertheless, its efficacy is highly dependent on the homogeneity of receptor expression. Tumor heterogeneity often results in the absence of receptors in certain pathological areas [28], creating ‘targeting blind spots.’ For bio-inspired carriers, such as cell-based delivery systems like CAR-T cells [29,30], although they possess inherent inflammatory tropism or antigen recognition capabilities, they are hindered by immunosuppressive factors and physical barriers within the tumor microenvironment [31], making deep infiltration into solid tumors challenging [32]. In the drug release stage, traditional responsive carriers mostly trigger release by responding to microenvironmental signals such as pH changes or enzyme concentration differences [33,34]. However, the physicochemical differences between normal and pathological tissues are often subtle. For instance, the pH difference between tumor tissue (5.5-6.5) and blood (7.4) is limited [35], which can easily lead to premature carrier degradation and drug leakage during blood circulation, or insufficient response at the target site, resulting in incomplete drug release.

**Scheme 1:**
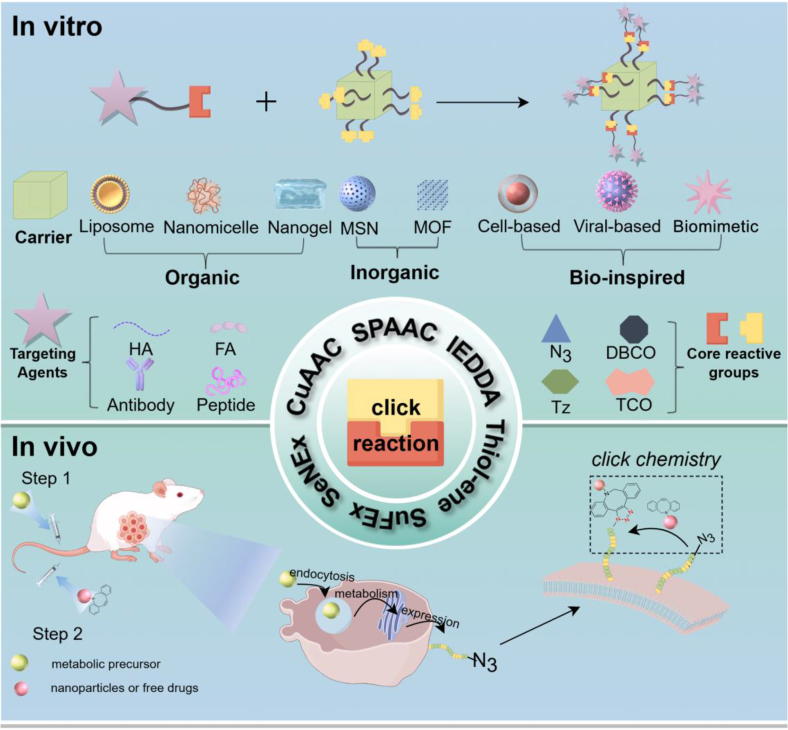
Click chemistry in the construction of actively targeted DDSs. In vitro Strategy: single click reaction for coupling targeting agents to carriers. In vivo Strategy: in vivo dynamic targeting based on click chemistry.

These limitations further give rise to a multitude of practical challenges. Firstly, the pharmacokinetic profile and biocompatibility of carriers are often imbalanced. Most nanocarriers are readily recognized and cleared by the reticuloendothelial system (RES) [36], necessitating PEGylation to prolong circulation time [37]. However, excessive PEGylation can also inhibit cellular uptake. While metal-based inorganic carriers (e.g., Fe_3_O_4_, AuNPs) exhibit high stability [38], they pose the risk of long-term accumulation in vivo, potentially leading to liver and kidney damage. Secondly, the synergistic effect of multi-drug co-delivery is often insufficient. Traditional carriers struggle to achieve precise proportional loading and synchronized release of drugs with different mechanisms of action, thus compromising the efficacy of combination therapy [[39], [40], [41]]. Thirdly, the technical limitations of functional modifications are significant. The conjugation efficiency between ligands and carriers is often less than 50% [42], and the conjugation process may compromise ligand activity or carrier structure [43]. For biomacromolecular drugs, such as nucleic acids and proteins, carrier modification must also consider drug stability and preservation of biological activity [44,45], posing significant technical challenges. Fourthly, there are scalability barriers in clinical translation. The carrier preparation process often generates isomers or byproducts, making batch-to-batch stability difficult to control [46]. Moreover, the large-scale synthesis of key materials like prodrugs and responsive linkers is expensive [47], further limiting the clinical adoption of the technology [48]. These issues collectively hinder the translation of conventional DDSs from laboratory research to clinical applications, necessitating breakthroughs through materials innovation and strategy optimization. Utilizing click chemistry reactions to address the aforementioned problems in drug delivery systems is highly effective. This review comprehensively discusses the applications of click chemistry reactions in the construction of active targeting drug delivery systems, with a primary focus on two strategies: the "In vitro Strategy: single click reaction for coupling targeting agents to carriers " and the "In vivo Strategy: in vivo dynamic targeting based on click chemistry," as shown in Scheme 1.

## 2 Overview of click chemistry reactions

In 2001, K. Barry Sharpless's team proposed “click chemistry,” a revolutionary chemical paradigm centered on enabling the facile construction of targeting agents by non-specialist chemists through highly efficient and reliable molecular joining reactions [49]. Analogous to the “buckle” mechanism of luggage straps, this concept emphasizes chemical coupling reactions characterized by high selectivity, high yield, mild reaction conditions, and ease of product isolation [50]. A core advantage is its ability to proceed efficiently in aqueous solutions, buffers, and even physiological environments without interfering with the functional groups of natural biomolecules [51], thus often being classified as an important branch of bioorthogonal chemistry [52,53]. These reactions do not require toxic catalysts or extreme reaction conditions, and certain copper-free click reactions further enhance reactivity and biocompatibility through designs incorporating ring strain [52,54]. This enables broad application in the precise chemical modification of live cell surfaces, cytoplasm, and even within living organisms in vivo. To summarize, we have outlined commonly used click chemistry reactions, as shown in Table 1.

**Table 1:** Commonly applied click chemistry reactions [[55], [56], [57], [58], [59], [60], [61], [62], [63], [64]].

Name	Reaction	K(M^−1^ s^−1^)	Advantages	Disadvantages	Applications
CuAAC		10-100	Fast reaction rate Suitable for unstable biomolecules	Cytotoxicity	Biomolecule Labeling and Imaging; Drug Delivery and Target Localization; Biomolecule Detection and Analysis
SPAAC		0.01-1	Free of catalyst	Reactivity with thiols	Biomolecule Labeling and Imaging; Biomolecule Detection and Analysis; Material Surface Modification and Device Fabrication
IEDDA		1-10^6^	Catalyst-free Excellent biocompatibility	The sensitivity of TCO to acids and the sensitivity of TZ to bases	Biomolecule Labeling and Imaging; Drug Delivery and Activation; Multicomponent Labeling and Dynamic Tracking
Thiol-ene		10^2^-10^3^	Strong functional group tolerance	Radical type requires initiator	Polymer Synthesis and Modification; Peptide and Biomolecule Modification; Surface and Materials Engineering
SuFEx		10^2^-10^4^	Modular	Some reactions require base catalysis	Drug design; Activity-based profiling; Protein targeting research; Assistance in bioorthogonal labeling
SeNEx		≥14.43	Strong functional group compatibility	Some reactions rely on metal catalysts	Org-Se Compound Synthesis; Bioconjugation; High-Throughput Drug Discovery; Carrier Optimization

### 2.1 CuAAC

Copper-catalyzed azide-alkyne cycloaddition (CuAAC) is an improved form of the classic Huisgen cycloaddition reaction [65,66]. It can proceed under near-physiological conditions through the action of copper catalysts, primarily generating 1,4-regioselective triazole products [67,68]. It exhibits rapid reaction kinetics (second-order rate constants can reach 4.6 × 10^5^ L/(mol·s)) [69,70], enabling conjugation within a short timeframe. This makes it particularly suitable for the modification of chemically or conformationally unstable biomolecules such as peptides [71], nucleic acids, proteins, and virus-like particles. In the field of drug delivery, it is widely employed for direct conjugation, antibody-drug conjugate (ADC) preparation, and gene drug carrier modification [72,73]. However, CuAAC has significant limitations. Copper catalysts exhibit considerable cytotoxicity [74], and even with ligand modulation, their interference with biological systems is difficult to completely eliminate [75,76]. This restricts its application in scenarios requiring extremely high biocompatibility, such as in vivo labeling of live cells and in vivo delivery [77]. Furthermore, CuAAC reaction shows high regioselectivity, predominantly producing the 1,4-triazole isomer with high product purity [78], which can reduce the purification cost and quality control difficulty in large-scale preparation [[79], [80], [81]].

Huang et al. [82] established a catalyst-free bioorthogonal addition reaction system between malononitrile and azodicarboxylate—the Malononitrile Azodicarboxylate Addition (MAAD) reaction. Utilizing malononitrile and azodicarboxylate as the reaction pair, this reaction proceeds rapidly and quantitatively in aqueous solution at room temperature and under physiological conditions without the need for external catalysts or additives, demonstrating excellent biocompatibility. Although this approach does not directly optimize CuAAC, it offers a highly effective alternative strategy for circumventing the issue of copper toxicity associated with that reaction.

### 2.2 SPAAC

Strain-promoted azide-alkyne cycloaddition (SPAAC) is one of the core reaction types within copper-free click chemistry [54]. By introducing ring strain into the alkyne structure, it can undergo efficient cycloaddition with azide groups under physiological conditions without the need for copper catalysts, generating stable triazole products [54,83]. Its reaction rate is moderate, demonstrating good aqueous compatibility and functional group tolerance [84,85]. This makes it suitable for various scenarios, including in vitro solution conjugation, live cell labeling [86], and in vivo drug delivery [87], while avoiding the copper toxicity issue associated with CuAAC. In drug delivery, SPAAC is widely used for carrier functionalization and targeted delivery. For instance, groups like dibenzocyclooctyne (DBCO) and BCN are modified onto the surface of nanocarriers such as liposomes, polymeric micelles [88], and metal-organic frameworks (MOFs) [47]. These then react with azide-modified targeting ligands (e.g., RGD peptides, folic acid, aptamers) or azide groups metabolically labeled on the surface of tumor cells, achieving active targeting and accumulation of drugs. It is also employed for site-specific modification of ADCs [[89], [90], [91]]. By reacting with azide-modified antibodies or drugs, it can produce ADCs with higher homogeneity, and the conjugation process has minimal impact on antibody binding activity. However, SPAAC has inherent limitations. Some reaction reagents have insufficient stability; for example, azide groups are prone to reduction, and BCN is susceptible to degradation under acidic conditions [54,92]. Hydrophobic cyclic structures of cyclooctyne derivatives like DBCO can lead to non-specific binding with blood proteins and aggregation, affecting the carrier's pharmacokinetics [93,94].

Jeong et al. overcame the core limitations of the SPAAC reaction by combining Signal Amplification by Reversible Exchange (SABRE) technology with benchtop Nuclear Magnetic Resonance (NMR): Addressing the challenge that SPAAC reactions in low-concentration systems are difficult to monitor in real time due to weak substrate signals and insufficient sensitivity of traditional NMR detection, they utilized SABRE technology to hyperpolarize azide or alkyne substrates in the reaction system. This significantly enhanced the NMR signal intensity of substrates at low concentrations, enabling real-time and precise tracking of the SPAAC reaction progress. Meanwhile, based on this real-time monitoring method, the reaction intermediates and byproduct formation trends can be dynamically captured, providing data support for optimizing reaction conditions (such as substrate ratio and reaction temperature) and effectively reducing the generation of byproducts. Ultimately, this improved the reaction controllability and efficiency of SPAAC in scenarios such as the synthesis of triazole-containing drugs [95].

### 2.3 IEDDA

The inverse electron-demand Diels-Alder (IEDDA) reaction, a hallmark of copper-free click chemistry [96], is characterized by its exceptionally fast reaction kinetics [92,97], primarily driven by the efficient coupling achieved through the pairing of tetrazines (Tz) with strained alkenes, such as trans-cyclooctene (TCO), or strained alkynes. This reaction exhibits second-order rate constants that can reach 10^3^-10^6^ L/(mol·s) [98], enabling efficient in situ reactions in vivo at low to mid-micromolar concentrations, without the need for catalysts, and demonstrating excellent biocompatibility [99,100]. Its versatility makes it highly applicable in drug delivery [51], with a diverse range of functionalities: It facilitates direct conjugation, such as active targeting achieved by reacting TCO- or tetrazine-modified carriers with targeting ligands [48,100]; its more critical role involves triggering drug release [101], where the tetrazine-TCO reaction, accompanied by chemical bond cleavage, allows for the release of chemotherapeutic drugs like doxorubicin (DOX) via mechanisms like allylic elimination or lactonization, or the release of carbonyl sulfide (COS) through the decomposition of thiocarbamates followed by conversion to hydrogen sulfide (H_2_S) signaling molecules [102]; and the construction of “click-release-fluorescence” systems for real-time drug release tracking [103].

Drawing on these advantageous properties, Y. Tian et al. [104]designed an active targeted drug delivery system based on the IEDDA click chemical reaction: the tetrazine-functionalized aggregation-induced emission photosensitizer TzPS5 was conjugated with cyclic Arg-Gly-Asp (cRGD) peptide to obtain TzPS5-cRGD, enabling it to actively target αᵥβ_3_ integrin highly expressed on the surface of tumor cells, and then combined with TCO-modified chemotherapeutic prodrug DOX-TCO. This system achieves in situ co-activation of the photosensitizer and prodrug via the IEDDA reaction, producing synergistic chemo-photodynamic therapeutic effects under light irradiation. It significantly inhibits the growth of primary and distant tumors, blocks pulmonary metastasis, reduces the toxic side effects of DOX used alone, and improves therapeutic safety.

Despite these advantages, IEDDA faces limitations: the reaction reagents lack sufficient stability, as tetrazines are prone to in vivo degradation by reducing agents, and TCO exhibits cis-trans isomerization issues [97]; some reaction systems rely on hydrophobic interactions for acceleration, leading to restricted controllability in complex biological environments [105].

### 2.4 Thiol-ene click reaction

The thiol-ene click reaction is a class of highly efficient click reactions employing thiol (-SH) and alkene (C=C) functional groups as reaction partners [106]. It proceeds primarily via a radical-mediated addition-chain transfer mechanism, though some variations can occur through nucleophilic addition pathways like thiol-Michael addition [107,108]. Its core characteristics include high yields, mild reaction conditions, insensitivity to oxygen and water, and excellent functional group tolerance [109]. The radical mechanism is often initiated by UV light or thermal initiators, where thiyl radicals undergo specific reactions with alkenes to form stable C-S-C bonds; electron-rich alkenes and strained alkenes exhibit significantly higher reactivity than electron-deficient alkenes [107]. Thiol-Michael addition, conversely, targets electron-deficient alkenes and proceeds rapidly under the catalysis of amine or phosphine bases. Recently, Chen et al. [110] employed a solvent-free, high-viscosity environmental control strategy to conduct a photoinitiated thiol-ene click reaction between a linear acrylate polymer bearing thiol side chains and a small-molecule diene containing a thioester group. This approach leveraged the restriction of segmental chain motion to suppress radical termination reactions between the polymer radicals, thereby extending their lifespan. In the fields of nanomedicine and materials science [111], this reaction finds extensive applications: it can be used for the preparation and crosslinking of polymeric carriers [112], such as constructing hydrogels and core-crosslinked nanomicelles through polymerization of thiols with vinyl monomers, or for surface functionalization of materials like poly(divinylbenzene) microspheres and cellulose nanocrystals to introduce targeting ligands, drugs, or imaging molecules. It also enables the conjugation of carriers with biomolecules [108], for instance, by linking thiol-modified proteins or peptides to alkene-functionalized nanocarriers, with minimal impact on the bioactivity of the biomolecules during the conjugation process.

Civril et al. [113] efficiently coupled the thiol-containing cyclic peptide ligand cRGDfC with a maleimide-terminated PEG-PLA block copolymer (Mal-PEG-PLA) via a thiol-maleimide click reaction. This successfully yielded polymeric micellar carriers targeting integrin receptors. This reaction, requiring no metal catalysts, proceeds under mild conditions with high specificity, enabling the precise linkage of the targeting ligand to the carrier in organic media. Following modification, the resulting carriers retained robust self-assembly capability and structural stability. The formed targeted carriers significantly enhanced cellular uptake efficiency in SKOV-3 ovarian cancer cells, which highly overexpress integrin receptors. Consequently, this improved uptake synergistically enhanced the antitumor activity of loaded agents, such as docetaxel and combretastatin A4, while maintaining high drug loading efficiency.

### 2.5 SuFEx

Sulfur(VI) Fluoride Exchange (SuFEx) is a new generation click chemistry reaction proposed by the Sharpless group in 2014 [114]. Utilizing sulfur(VI) fluorides as the core reactive unit, it achieves efficient molecular coupling through the selective exchange of sulfur-fluorine (S-F) bonds [115]. Key features include modularity, mild reaction conditions, high yields, and broad functional group tolerance [116]. Notably, it requires no metal catalysts and exhibits excellent biocompatibility [117]. The reaction mechanism relies on the activation and departure of fluoride ions under specific conditions, often catalyzed by bases such as triethylamine, DBU, or difluorides [118]. Molecular linkages are formed through stable covalent bonds, including sulfur-oxygen (S-O) and sulfur-nitrogen (S-N) bonds. Some reactions can achieve high-level second-order rate constants. Within the SuFEx reaction system, a diverse array of “molecular plug-ins” is available, including sulfonyl fluorides (R-SO_2_F), sulfuryl fluoride (SO_2_F_2_), thionyl tetrafluoride (SOF_4_), vinyl sulfonyl fluoride (ESF), and 1-bromoethyl sulfonyl fluoride (BESF) [119,120]. Among these, SOF_4_ can construct three-dimensional products, overcoming the limitations of traditional planar linkages, while ESF combines SuFEx reactivity with Michael addition activity, enabling multiple functionalizations [121]. In the biomedical field (Bio-SuFEx), sulfonyl fluoride probes are employed for protein labeling and activity analysis [122]. Fluorosulfate derivatives can enhance drug activity, and precise modification of ADCs is achievable [123].

Beyond its application in constructing precisely modifiable drug delivery systems—where Hoveyda et al. [124] developed a click chemistry strategy based on bisphosphine-copper-catalyzed phenoxydiazaborinine formation (CuPDF) and copper-palladium-catalyzed quinoline formation (Cu/PdQNF), and synergistically combined it with the SuFEx reaction to construct an active targeted drug delivery system. In this system, CuPDF enables the ligation and in situ modification of cyano-containing substrates with drugs and targeting moieties, generating tunable fluorescent linkers; Cu/PdQNF accomplishes modification in aqueous media, and both are orthogonal to CuAAC and SuFEx. The system can synthesize single-drug and dual-drug conjugates targeting cyclic peptides (such as cilengitide analogs). The fluorescent linkers allow tracking of the delivery pathway, and chemoselective modification capabilities enable precise drug conjugation and release, enhancing the targeting and therapeutic precision of the delivery system.

### 2.6 SeNEx

The Selenium-Nitrogen Exchange (SeNEx) reaction is a novel click chemistry modality inspired by the biochemical reaction between Ebselen and cysteine residues [55]. This reaction centers on an electrophilic Selenium(II) species as the core reaction unit, facilitating flexible and efficient molecular conjugation through the selective exchange of the Se–N bond, resulting in the formation of a stable Se–C bond. Key characteristics of SeNEx include modularity, predictability, rapid kinetics (second-order rate constants, k_2_ ≥ 14.43 M^−1^ s^−1^),mild reaction conditions, and excellent functional group tolerance. While catalysts such as Silver(I) or Copper(I) can be employed, metal-free catalytic variants with good biocompatibility have also been developed [56]. The SeNEx reaction system accommodates various “molecular plugs,” including selenium sources such as benzoselenazolone (BSEA) and benzothiaselenazol-1-oxide (BTSA), which react with different nucleophiles to form C (sp^2^)-Se or C (sp)-Se bonds. In the biomedical field, SeNEx enables the nanomolar-scale parallel synthesis of selenium-containing natural product libraries and DNA-encoded libraries (SeDELs). It is widely utilized in the late-stage modification and ligation of peptides, multifunctionalization of proteins, and the synthesis of seleno-macrocycles, thereby providing a powerful tool for drug discovery and chemical biology research [57].

## 3 Click chemistry in the construction of actively targeted drug delivery systems

Click chemistry is utilized for the construction of active targeting DDSs through in vitro and in vivo strategies. The in vitro strategy utilizes organic, inorganic, and bio-based carrier materials as platforms. Click reaction groups such as azides (N_3_) are modified onto their surfaces, while complementary click groups like DBCO and TCO are conjugated to targeting agents, including targeting ligands and drug precursors. Click chemistry is then employed for the efficient coupling of the carrier with these targeting agents. The in vivo strategy relies on the synergistic action of metabolic engineering and click chemistry. First, bioorthogonal click group precursors, such as N_3_, are introduced into the body via local injection or systemic delivery mediated by nanocarriers. These precursors are integrated into cell membrane structures of target cells (e.g., cancer cells, bacteria) through their inherent metabolic pathways, enabling specific display of bioorthogonal groups on the target cell surface. Subsequently, drug-loaded nanocarrier systems modified with complementary click groups are administered intravenously. Through copper-free click reactions (e.g., SPAAC, IEDDA), covalent bonds are formed between the nanocarrier system and the bioorthogonal groups on the target cell surface, achieving drug enrichment and retention at the target site. This effectively addresses issues in traditional delivery systems, such as poor tumor penetration and off-target distribution.

### 3.1 In vitro strategy: click reaction for coupling targeting agents to carriers

Utilizing diverse carrier materials as platforms, click-reactive groups such as N_3_ are modified onto their surfaces. Simultaneously, complementary click groups (e.g., DBCO, TCO, TZ) are conjugated to targeting agents, including targeting ligands and drug precursors. Click chemistry is then employed to achieve efficient coupling between the carrier material and the targeting agents. The selection of targeting agents is dictated by the intended application; for instance, targeting agents can impart tumor or specific organ targeting capabilities to carriers, while drug precursors enable in situ assembly of active drugs at the disease site. Commonly used targeting agents encompass diverse types, including natural polysaccharides, vitamin derivatives, bioactive polypeptides, and specific antibodies. Among these, hyaluronic acid (HA), folic acid (FA), polypeptides (e.g., RGD peptide, cell-penetrating peptides CPPs), and antibodies (e.g., anti-HER2 antibody, anti-EGFR antibody) have emerged as key targeting agents with significant clinical translation potential due to their high specificity, good biocompatibility, and well-defined targeting mechanisms. Optimization of the click group ratio and reaction conditions is crucial to balance coupling efficiency, carrier stability, and targeting agent activity. Ultimately, this approach yields DDSs that possess targeting ability, stability, and biocompatibility, laying the foundation for applications such as precision drug delivery or protein degradation.

#### 3.1.1 Organic material carrier

DDSs based on organic materials have significantly improved drug targeting efficiency and clinical applicability through the innovative design of biocompatible and biodegradable materials [125,126]. These systems are primarily based on core materials such as liposomes, nanomicelles, and nanogels. Through material functionalization and structural optimization, these systems have addressed limitations associated with traditional drug delivery, such as poor permeability, short half-lives, and systemic toxicity, and have been widely applied in areas including cancer chemotherapy, gene therapy, and diagnostic imaging, with ongoing efforts to advance towards intelligent and multifunctional designs [[127], [128], [129], [130], [131]]. When employing click chemistry with organic carriers such as liposomes or nanomicelles, critical considerations include the aggregation of carriers and non-specific binding to blood proteins resulting from the hydrophobicity of the click reagents [132], as well as the compatibility between or enzyme-responsive linkers and the click reaction itself [133], which must be managed to prevent compromising drug encapsulation efficiency and in vivo circulation stability [134]. In general, this review synthesizes the content of constructing active targeting DDSs by conjugating targeting agents to organic carrier via click chemistry reactions, as shown in Table 2.

**Table 2:** Summary of click chemistry in Organic Carrier.

Type of DDSs	Click reaction	Deliver the load	Targeted area	Purpose	Ref
Liposome	CuAAC	siRNA HA	Subcutaneous tumor	Covalent attachment of HA to the surface of cationic liposomes	146
	SPAAC	Glycophorin A(GPA)antibody PECAM antibody ICAM-1 antibody	Endothelial cells RBCs	The two antibodies are covalently attached to the liposome	147
	IEDDA Thiol-maleimide	ALK ligand precursor (W4) CRBN ligand precursor (Z2) Targeting peptide cRGD	Subcutaneous tumor	Realization of in vivo in situ assembly of PROTACs	148
Nanomicelle	Thiol-maleimide	Cisplatin Thiolated Collagenase	Subcutaneous tumor	Attachment of thiolated collagenase to maleimide moieties on the surface of nanocarriers	154
	CuAAC	IR780 iodide	Subcutaneous tumor	Building molecular brushes with precise structures	155
	Thiol-ene	DOX	Subcutaneous tumor	Introduction of functional groups into the main chain of PJL polymers	156
	Amine-epoxy	PTX Ce6	Subcutaneous tumor	Construction of the main chain structure of the polymer HBPMT	157
Nanogel	Thiol-ene Michael Addition	FITC - Dextran		Construction of photoresponsive hydrogel networks	163
	Thiol-ene	CIP NB	Wound area	Building hydrogel networks	164
	CuAAC	DOX cRGD peptide	Glioblastoma	Formation of nanogels from functionalized PVA through chemical bonding	166
	SPAAC	DOX	Subcutaneous tumor	Constructing crosslinked networks for nanogels	167
	Thiol-ene	MET DOX	Colorectal cancer tumor tissue	Cross-linking of HA-SH, SA-SH and PEG-acr	168

##### 3.1.1.1 Liposome

Liposomes are spherical, artificial vesicles composed of a lipid bilayer, capable of encapsulating both hydrophilic and hydrophobic substances [135]. Their basic structure comprises an aqueous core for drug encapsulation and a lipid bilayer composed of phospholipid molecules, with the hydrophobic tails oriented inward and the hydrophilic heads oriented outward, forming a stable vesicular structure [136,137]. Liposome-based drug delivery systems effectively protect drugs from the influence of the in vivo environment, enhancing drug stability and bioavailability [10,138]. They achieve passive targeting through enhanced permeability and retention effect in tumor vasculature, or active targeting by conjugating targeting molecules such as antibodies or folic acid to facilitate receptor-mediated recognition on tumor cell surfaces, enabling targeted drug delivery, reducing drug distribution in non-target tissues, and lowering toxicity [[139], [140], [141], [142], [143], [144]]. Click chemistry can be employed for the surface modification and targeting ligand conjugation of lipid nanoparticles through various highly efficient and specific reactions. Furthermore, it can be synergistically combined with combinatorial chemistry and barcoding techniques to optimize formulations and enhance the efficacy of RNA delivery [145].

Mo et al. [146] employed the CuAAC reaction as the core strategy. They used azide-modified cholesterol (Chol-N_3_) and alkyne-modified hyaluronic acid (alk-HA) as click modules. Initially, cationic liposomes and siRNA were physically assembled through electrostatic interactions to form RSC complexes. Subsequently, the alk-HA was chemically conjugated to the surface of the RSC complexes via click reaction to yield RSC-HA nanocarriers. HA serves as an active targeting ligand, specifically binding to the overexpressed CD44 receptors on the surface of tumor cells, thereby enabling targeted delivery to tumors. Concurrently, the chemically conjugated HA effectively shields the positive charge of the nanocarrier, enhancing blood circulation stability. Moreover, it can be synergistically degraded by tumor cell-associated hyaluronidase (HAase) and glutathione (GSH), triggering siRNA release to amplify gene silencing efficacy. This approach effectively addresses the trade-offs between cationic carrier toxicity, siRNA binding efficiency, and stability.

**Fig. 1:**
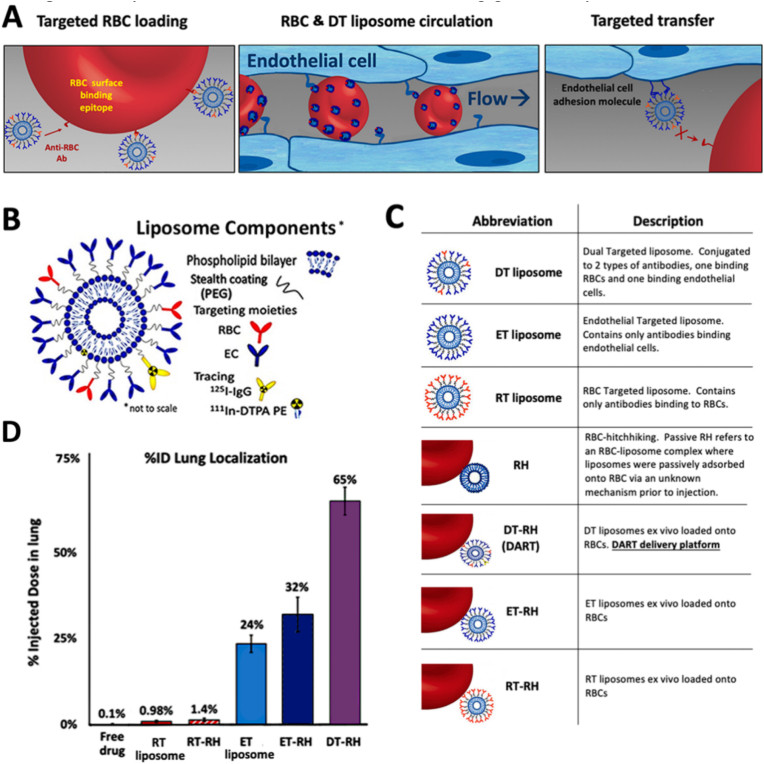
DART more than doubles the efficiency of organ-targeting compared to targeting via affinity-ligands-only and RBC hitchhiking. (A) Goal mechanism of DART. (B) DART liposome components. (C) Nomenclature for DART, predicate technologies, and controls. (D) The percentage of injected dose (% ID) of different types of liposomes. Reprinted with permission from Ref. [147]. Copyright 2022 American Chemical Society.

Ferguson et al. [147] developed the DART (Dual-Affinity Red blood cell and Target cell) active targeting drug delivery system. Its construction relies on SPAAC click reaction: azide-functionalized PEGylated liposomes were coupled with DBCO-modified monoclonal antibodies (targeting red blood cell GPA and lung endothelial cells PECAM/ICAM, respectively) to produce dual-targeted liposomes (DTs) (Fig. 1B and C). Following intravenous administration via red blood cell hitchhiking (RH) in mice, the anti-RBC antibody initially binds to erythrocytes. Upon reaching the pulmonary capillary bed, the endothelial-targeting antibody binds to target cell epitopes, facilitating liposome transfer (Fig. 1A). This system achieved 65% injected dose accumulation in the lungs within 30 min in mice, which is 650-fold higher than free drugs and over 2-fold higher than the ET-RH system (Fig. 1D). Furthermore, its targeting selectivity to endothelial cells was 6-fold greater than that of localized leukocytes. Efficient targeting was also observed in an ex vivo human lung perfusion model, with no significant complement activation and no apparent abnormalities in cardiopulmonary function or tissue structure, indicating good safety.

**Fig. 2:**
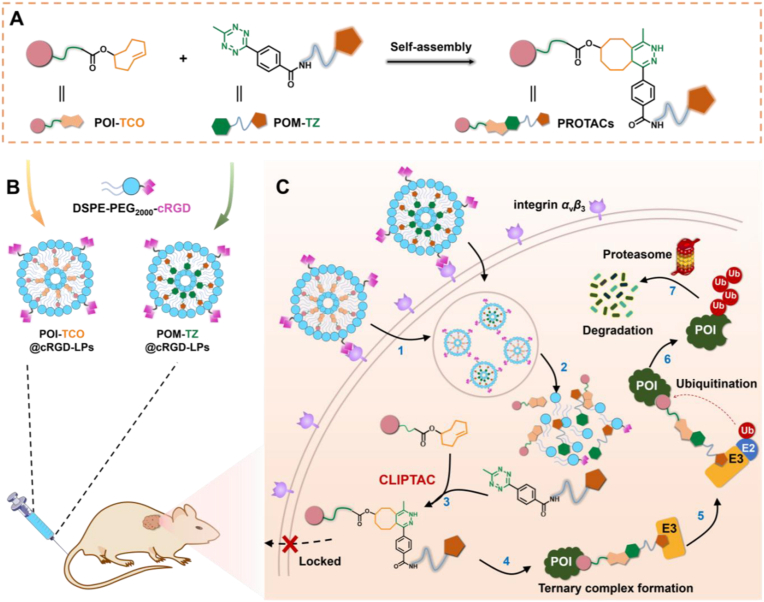
Schematic illustration of the Nano-CLIPTACs strategy for tumor specific protein degradation. (A) Bioorthogonal reaction-enabled self-assembly. (B) Preparation scheme of POI-TCO@cRGD-LPs and POM-TZ@cRGD-LPs. (C) Proposed degradation pathway of POI from Nano-CLIPTACs therapy in vivo*.*Reprinted with permission from Ref. [148]. Copyright 2024 Wiley.

Xie et al. [148] developed the Nano-CLIPTACs active targeting drug delivery system, which integrates click chemistry and targeted delivery technologies. First, traditional PROTACs were disassembled into a TCO-containing ALK ligand precursor (W4) and a TZ-modified CRBN ligand precursor (Z2). These precursors were then assembled in situ via an IEDDA click reaction (Fig. 2A). Subsequently, both precursors were individually encapsulated within cRGD-modified liposomes (Fig. 2B). Active targeting was achieved through the specific binding of cRGD to the highly expressed integrin αvβ_3_ on tumor cell surfaces (Fig. 2C). In H3122 cells, this system efficiently assembled into functional PROTACs (WZ42), achieving an ALK degradation DC_50_ of 175.37 ± 53.24 nM without exhibiting a ‘hook effect.’ In animal models, the concentration of WZ42 at tumor sites was 30-fold higher compared to the non-encapsulated group, resulting in a tumor growth inhibition rate of 77.4%. Furthermore, the system demonstrated low toxicity to normal organs, indicating good safety.

##### 3.1.1.2 Nanomicelle

**Fig. 3:**
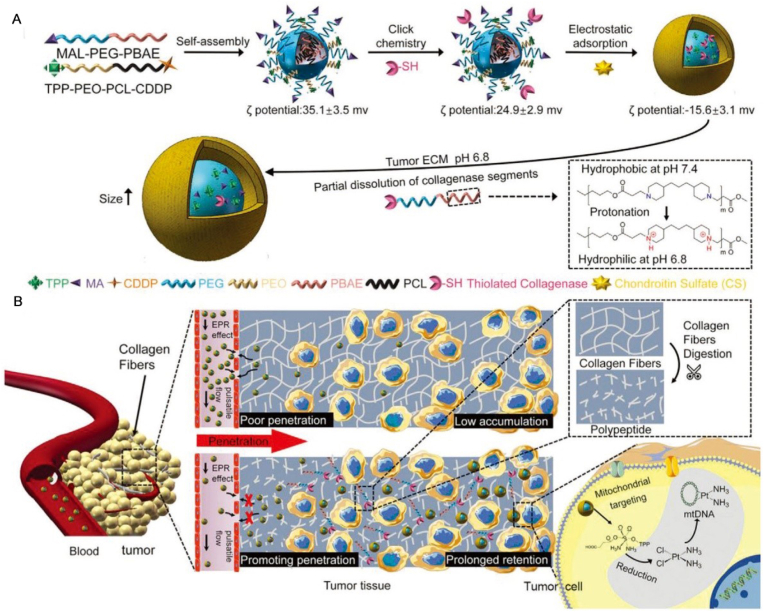
The size-changeable collagenase-modified nanoscavenger prompting penetration and retention of nanomedicine in deep tumor tissue. (A) Fabrication of CS/Col-TCPPB NPs, as well as the size of the NPs increased and the collagenase containing components was dissolved out in response to the acidic pH. (B) Schematic illustration of the accumulation of NPs in tumor. In deep tumor tissues, under the combined action of collagenase digestion of collagen fibers and particle size increasing, the penetration and retention of nanomedicines are increased significantly; in tumor cells, the NPs can specifically target mitochondria and release the cisplatin drugs into mitochondria, causing a destruction of mitochondrial DNA. Reprinted with permission from Ref. [154]. Copyright 2020 Wiley.

Nanomicelles are core-shell structured nanoparticles formed by the self-assembly of amphiphilic block copolymers [149]. Their hydrophobic core can efficiently encapsulate poorly water-soluble drugs, such as paclitaxel and docetaxel, while the hydrophilic shell extends blood circulation time and prevents immune clearance through steric hindrance [[150], [151], [152]]. Click reactions enable the efficient conjugation of a wide variety of targeting ligands at distinct sites on the micelle surface (e.g., chain ends, side chains, or post-micelle self-assembly) [153]. This process simultaneously preserves both the biological activity of the conjugated ligands and the structural integrity of the micelles. Xu et al. [154] developed a size-tunable collagenase-modified nano-scavenger (CS/Col-TCPPB NPs) for active targeting drug delivery. Its construction utilizes ‘click’ chemistry: first, TCPPB micelles with maleimide end-groups were formed via self-assembly. Subsequently, a ‘click’ reaction between thiol-modified collagenase and the maleimide groups on the micelle surface was employed for conjugation, followed by encapsulation with chondroitin sulfate to obtain the system (Fig. 3A). This system protects collagenase activity via chondroitin sulfate (Fig. 3B). In the acidic tumor microenvironment, some collagenase-containing components dissolve and degrade collagen fibers to enhance penetration. The remaining components cause the nanoparticles to swell to approximately 250 nm, thereby increasing retention. Concurrently, it utilizes TPP for targeted mitochondrial release of cisplatin. In a 4T1 tumor model, platinum (Pt) accumulation at the tumor site reached 15.03 ± 0.83 % ID/g, which is 20.2-fold higher than free cisplatin, resulting in a tumor growth inhibition rate of 94.6%. Furthermore, no significant hepatorenal toxicity was observed, indicating good safety.

Li et al. [155] constructed a morphology-tunable molecular brush (MBB) active targeting drug delivery system using CuAAC click chemistry. The system features a poly(2-hydroxyethyl methacrylate) (PHEMA) backbone containing alkyne groups. Through a click reaction, azide-modified PEG-b-PtBA (poly(ethylene glycol)-block-poly(tert-butyl acrylate)) side chains were coupled to the backbone. Subsequent hydrolysis yielded core-shell unimolecular micelles with spherical, rod-like, or worm-like morphologies, used for loading the photothermal agent IR780. Among these, the rod-like MBB (B122-IR780) demonstrated optimal performance, achieving an IR780 loading capacity of approximately 25% and maintaining an unimolecular state without aggregation in media containing serum. It exhibited superior cellular uptake efficiency by MCF-7 cells and enhanced penetration into 3D spheroids in vitro. In vivo, tumor accumulation of the rod-like MBB was significantly higher than that of the spherical and worm-like MBBs and free IR780. Upon irradiation with an 808 nm laser, the tumor temperature reached 55.6 °C, effectively inhibiting tumor growth. Furthermore, no significant toxicity to major organs was observed.

**Fig. 4:**
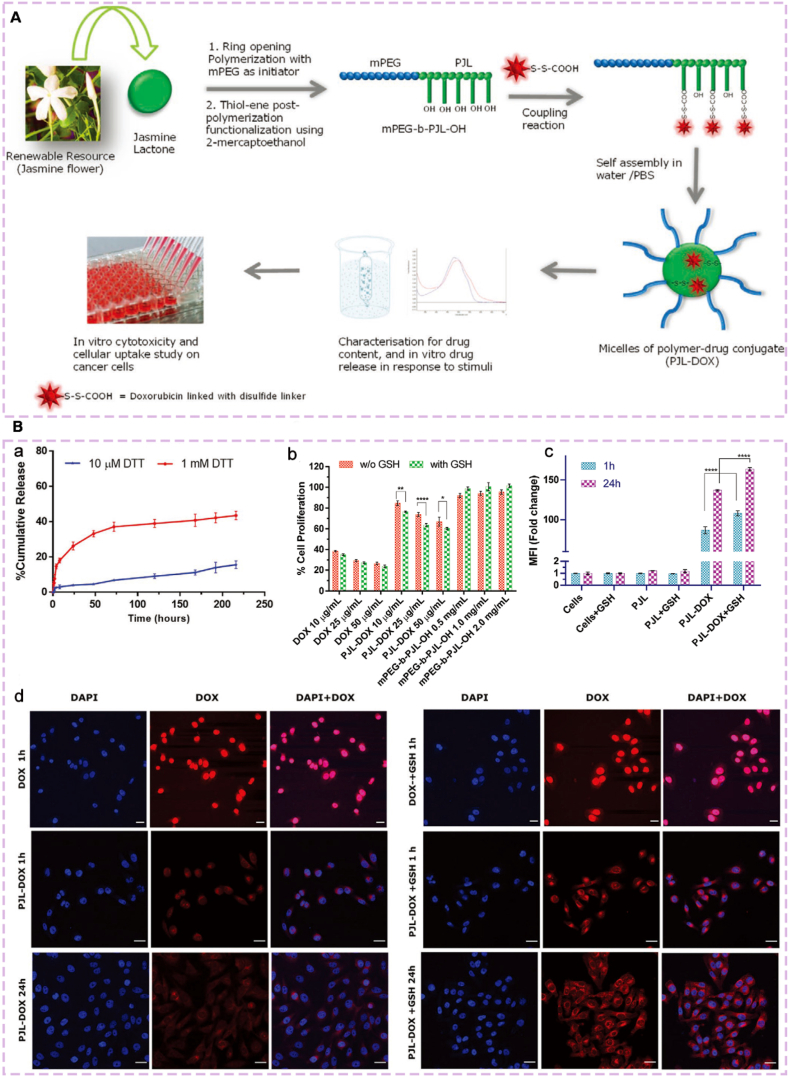
(A) Schematic diagram of the preparation and overall evaluation of stimuli-responsive DDS. (B) Functional verification experiments of micelles. (a) Reduction-responsive drug release curve. (b) Cell proliferation inhibition experiment. (c) Cytotoxicity (MF value) detection. (d) Cell Uptake and Drug Distribution Imaging. Reprinted with permission from Ref. [156]. Copyright 2021 Wiley.

Bansal et al. [156] constructed a stimulus-responsive drug delivery system based on renewable jasminellalide using UV-mediated thiol-ene click chemistry. First, a thiol-ene responsive amphiphilic block copolymer, mPEG-b-PJL (poly(ethylene glycol)-block-poly(jasminellalide)), was synthesized via ring-opening polymerization, yielding a polymer with olefinic functionalities. Subsequently, thiol-containing compounds were modified onto the copolymer backbone through a click reaction, producing derivatives with functional groups such as hydroxyl and carboxyl groups. The hydroxyl-modified product, mPEG-b-PJL-OH, was then coupled with DOX via a redox-responsive disulfide bond to obtain PJL-DOX, which self-assembled into micelles approximately 150 nm in size (Fig. 4A). This system triggered DOX release in tumor cells (MDA-MB-231) in the presence of 10 mM GSH. After 24 h of incubation, fluorescence intensity significantly increased, and cytotoxicity was markedly enhanced (Fig. 4B). The carrier itself exhibited good biocompatibility, providing an effective platform for precise tumor therapy.

**Fig. 5:**
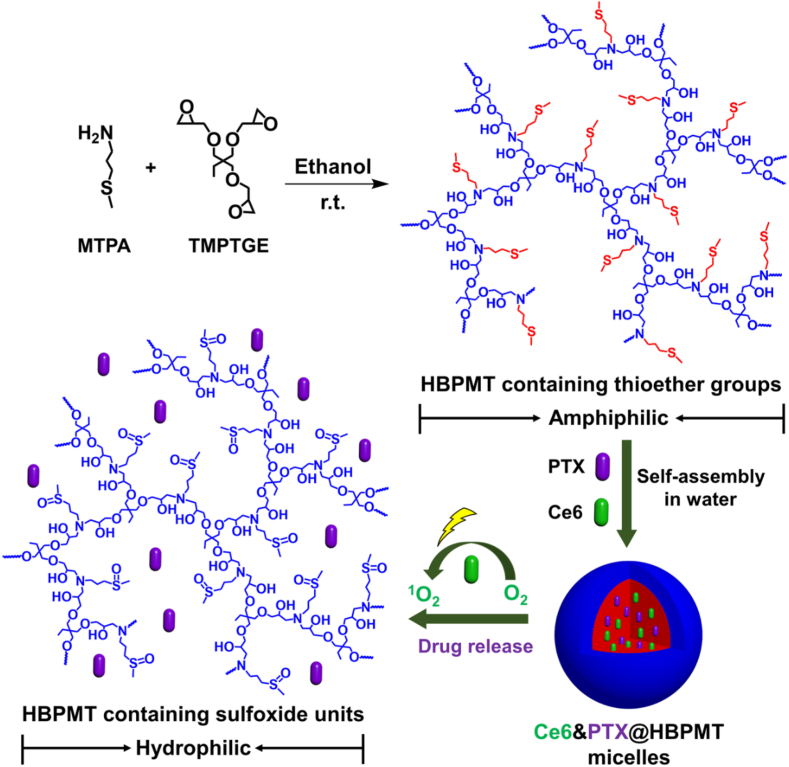
Synthetic route of amphiphilic hyperbranched polymer HBPMT and schematic illustration for preparation of (photosensitizer and drug)‐loaded micelles (Ce6&PTX@HBPMT) and light‐triggered drug release. Reprinted with permission from Ref. [157]. Copyright 2022 Wiley.

Wang et al. [157] utilized amine-epoxy click chemistry to synthesize a sulfur-containing amphiphilic hyperbranched polymer (HBPMT) from monomers MTPA and TMPTGE. This polymer self-assembled into stable micelles for co-loading of PTX (paclitaxel) and Ce6 (chlorin e6) (Fig. 5). This constructed a ROS-responsive DDSs. Under 660 nm laser irradiation, Ce6 generates reactive oxygen species (ROS) that oxidize the thioether linkages, triggering micelle disassembly. After 24 h, PTX release reached 74.8%, exhibiting significant inhibition of MCF-7 cell proliferation. This achieved synergistic chemotherapy-photodynamic therapy, and the carrier demonstrated good biocompatibility.

##### 3.1.1.3 Nanogel

Nanogels are cross-linked hydrogel particles with nanoscale dimensions (typically 20-250 nm), characterized by high water content, tunable size, large surface area, and abundant space for accommodating bioactive molecules [158]. Their small size, good biocompatibility, and tunable release properties make nanogels ideal carriers for drug delivery [[159], [160], [161], [162]].

**Fig. 6:**
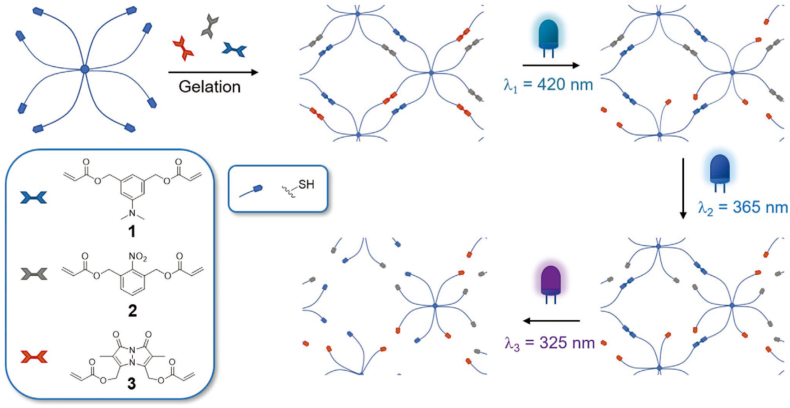
Schematic of wavelength-selective hydrogel softening: 8-arm PEG-thiol as matrix, three chromophores (aminobenzene, o-NB, bimane; 320/365/420 nm-responsive) as acrylate linkers, gelled via Michael addition and stepwise degraded by specific wavelengths. Reprinted with permission from Ref. [163]. Copyright 2021 American Chemical Society.

Pelloth et al. [163] constructed a wavelength-selective responsive hydrogel drug delivery system using Michael thiol-ene click chemistry. An 8-arm PEG-thiol served as the matrix. Three photo-sensitive chromophores with acrylate groups, each absorbing at specific wavelengths (320 nm for DMAB, 365 nm for o-NB, and 420 nm for benzil), were used as crosslinkers. These were crosslinked via click reaction to form a hydrogel network. This system can sequentially degrade crosslinking bonds upon irradiation with specific wavelengths of light, enabling stepwise softening of the hydrogel and mesh size control (increasing from approximately 5 nm to 8 nm) (Fig. 6). Furthermore, mechanical properties can be precisely tuned based on the chromophore ratio. The material and its photodegradation process exhibited extremely low cytotoxicity to pre-osteoblast cells (viability >90%). It can also modulate cell spreading behavior through softening, providing an efficient platform for precise drug release and biomaterial engineering.

**Fig. 7:**
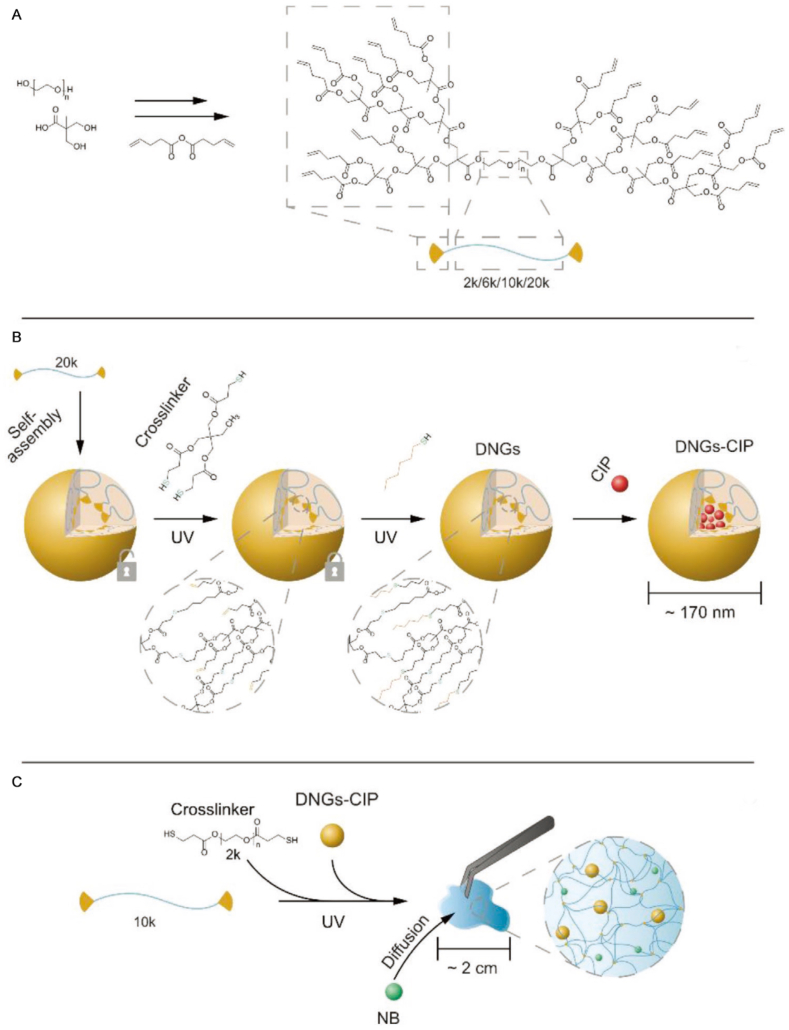
Schematic of synthesis: (A) Allyl-terminated HBDLDs; (B) Post-functionalized dendritic nanogels (DNGs) with CIP loaded in hydrophobic cores; (C) Hybrid hydrogels containing hydrophobic/hydrophilic antibiotics. Reprinted with permission from Ref. [164]. Copyright 2020 Wiley.

Fan et al. [164] constructed a dendrimeric hybrid hydrogel drug delivery system for antibiotic co-delivery using thiol-ene click chemistry. First, allyl-functionalized hyperbranched dendrimer-linear-dendrimer copolymers (HBDLDs) were crosslinked with a thiol crosslinker via a click reaction to prepare dendritic nanogels (DNGs) loaded with the hydrophobic antibiotic ciprofloxacin (CIP) (Fig. 7A). Subsequently, these DNGs-CIP were combined with HBDLDs and thiol-terminated PEG, followed by UV-curing via click reaction to form a hybrid hydrogel (Fig. 7B). Finally, hydrophilic antibiotic neomycin sodium salt (NB) was loaded by diffusion (Fig. 7C). The system's elasticity is tunable (2-14.7 kPa, matching human skin). It achieved rapid release of NB (over 87% released within 4 h) and sustained release of CIP (reaching maximum release at 48 h). In an in vitro infection model, bacterial reduction in HaCaT and human dermal fibroblast cells reached 99% and 97%, respectively. The resulting antibacterial wound dressing exhibited superior antibacterial activity compared to commercial products, promoted skin cell proliferation, and degraded into non-toxic components, demonstrating excellent biocompatibility. Shen et al. [165] similarly utilized reversible addition-fragmentation chain transfer (RAFT) polymerization technology to prepare functional polymers, subsequently constructing biocompatible composite nanosystems through the interaction between the polymers and nanomaterials.

Wei Chen et al. [166] constructed a cyclic RGD-modified, reduction-responsive, active targeting drug delivery system utilizing CuAAC-like click chemistry. Starting with carboxyl/alkyne-functionalized and azide-functionalized PVA (poly(vinyl alcohol)), they prepared disulfide-containing nanogels (SS-NGs) via an inverse suspension precipitation method in acetone using propargyl alcohol as a terminator and click reaction. Subsequently, cyclic RGD peptides were coupled to obtain cRGD-SS-NGs. This system can target U87-MG cells overexpressing αᵥβ_3_ integrins and rapidly release drugs under intracellular stimuli. In vivo, tumor uptake reached 5.54% ID/g (8-fold higher than free DOX), effectively inhibiting tumor growth with lower side effects.

Gregor Nagel et al. [167] constructed a matrix metalloproteinase (MMP)-sensitive, multi-stage nanogel active targeting drug delivery system using SPAAC click chemistry. A polyglycerol dendrimer with bicyclononyne (BCN) groups (dPG-BCN) served as the backbone. This was crosslinked with an MMP-sensitive fluorescent peptide crosslinker bearing azide groups at both ends via a SPAAC reaction, followed by inverse suspension precipitation to prepare peptide crosslinked nanogels (pNGs). Subsequently, DOX was coupled via a pH-sensitive linker to obtain pNG-DOX. This system, upon degradation by MMPs in the tumor microenvironment, reduced in size from over 200 nm to below 50 nm. Its penetration depth in agarose gels reached 8.9 mm (compared to only 2.5 mm for the non-degraded group), demonstrating significantly superior penetration in multicellular tumor spheroids compared to the non-degraded control.

group. It efficiently delivered DOX to deep tissue, reducing tumor spheroid ATP content to 22% of the control group, thereby achieving precise and efficient tumor drug delivery.

**Fig. 8:**
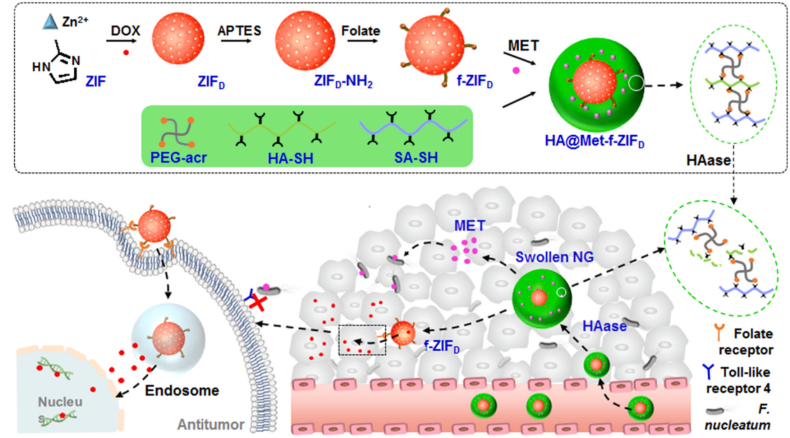
Illustration of the preparation process and antibacterial/anticancer mechanism of HA@Met-f-ZIF_D_ NGs. Reprinted with permission from Ref. [168]. Copyright 2023 Elsevier.

Shuang Xie et al. [168] constructed a size-tunable, active targeting drug delivery system using thiol-ene click chemistry (Fig. 8). Using thiol-modified hyaluronic acid (HA-SH) and thiol-modified sodium alginate (SA-SH) along with 4-arm polyethylene glycol acrylate (PEG-Acr) as raw materials, these were crosslinked via the click reaction in an inverse miniemulsion. This resulted in nanogels (HA@Met-f-ZIF_a_) loaded with DOX-carrying folate-modified zinc imidazole framework (f-ZIF_a_) and metronidazole (MET). In the tumor microenvironment, the system expands in size from 200 nm to 1500 nm upon degradation by hyaluronidase, prolonging retention. The gradually released f-ZIF_a_ mediates tumor cell targeting via folate, and drugs are released under acidic conditions. The system extends the half-lives of MET and DOX by approximately 20-fold. DOX content in tumors was 6-fold higher than that of free drug, effectively clearing *Fusobacterium nucleatum* and inhibiting tumor growth. Mouse survival was significantly prolonged, with no apparent hepatorenal toxicity.

#### 3.1.2 Inorganic carrier

DDSs based on inorganic materials utilize inorganic nanomaterials, such as metal-organic frameworks (MOFs) and Mesoporous Silica Nanoparticles (MSNs), as drug carriers due to their excellent physicochemical properties and biocompatibility. These nanocarriers enable effective drug delivery through various mechanisms, including passive and active targeting [169]. The high surface area and tunable pore structure of inorganic nanomaterials allow for efficient drug loading and controlled release [170,171]. Furthermore, these systems exhibit good stability and resistance to degradation, enabling them to maintain drug activity in vivo for extended periods, and are widely used in fields such as cancer therapy, gene therapy, and vaccine delivery [172]. Inorganic carriers, such as MOFs and MSNs, require attention to the influence of click reactions on their porous structure and surface active sites [173]. By optimizing the design of inorganic materials, researchers can improve drug bioavailability and targeting, thereby enhancing therapeutic efficacy and reducing side effects. In general, this review synthesizes the content of constructing active targeting drug delivery systems by conjugating targeting agents to inorganic carrier via click chemistry reactions, as shown in Table 3.

**Table 3:** Summary of click chemistry in Inorganic Material Carrier.

Type of DDSs	Click reaction	Deliver the load	Targeted area	Purpose	Ref
MOFs	CuAAC	DCA Calcein	Subcutaneous tumor	Covalent attachment of PEG chains to the surface of UiO-66 nanoparticles	179
	CuAAC	Resveratrol (Rsv) analogs	Mitochondria in tumor cells	In situ drug synthesis in mitochondria within tumor cells	180
	CuAAC	DOX	Subcutaneous tumor	Synthesis of DEX-ALN-PEG copolymers	181
	CuAAC	Photosensitizer (PS) precursor	Mitochondria in tumor cells	In situ synthesis of cancer cell-specific PS	182
MSNs	Thiol-maleimide	DOX	Subcutaneous tumor	Attachment of thiolated collagenase to maleimide moieties on the surface of nanocarriers	186
	Thiol-ene	Curcumin	Subcutaneous tumor	Construction of polymer-drug couplings with pH sensitivity	187
	IEDDA	Anti-CD11b antibody	CD11b^+^ cells	Achieve efficient binding of nanoparticles to immune cells	188
	Thiol-ene	DOX Antimicrobial peptide HHC36	Lung tumor cells	Fix the AMP on the surface of the MSNs	189

##### 3.1.2.1 MOF

MOFs are porous crystalline frameworks characterized by precisely controlled structures, immense diversity, and high porosity [174]. Owing to their well-defined crystalline structures and inherent porosity [175], MOFs are considered excellent templates for creating novel MOF-based composite materials. Likewise, their unique physicochemical properties make them particularly suitable for drug delivery applications, especially in targeted delivery and controlled release [[176], [177], [178]].

**Fig. 9:**
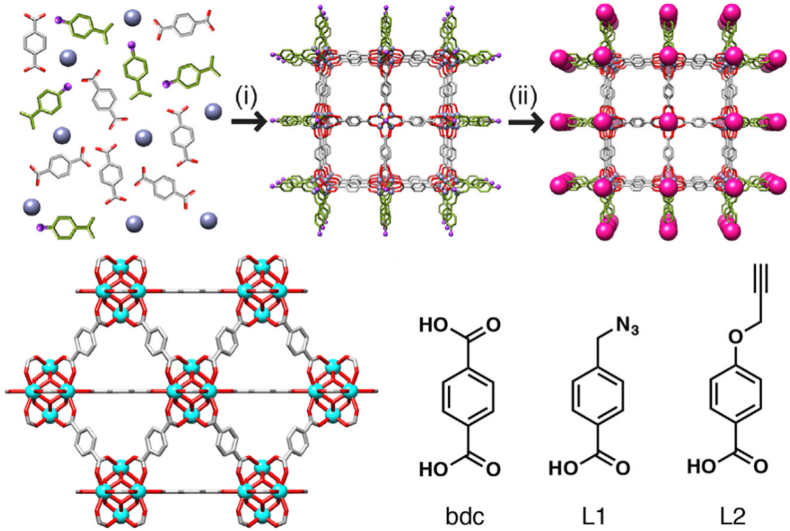
Click Modulation of UiO-66 MOFs. Reprinted with permission from Ref. [179]. Copyright 2017 Elsevier.

Forgan et al. [179] constructed a pH-responsive, active targeting drug delivery system using CuAAC click chemistry. Using UiO-66 nanoparticles functionalized with an azide-containing modulator (L1) as the carrier, alkyne-modified polyethylene glycol (PEG) was covalently grafted onto their surface via CuAAC reaction, yielding PEGylated UiO-66 nanocarriers (Fig. 9). This system exhibited enhanced stability under pH 7.4 conditions, with less than 30% drug release within 5 days, thus avoiding a ‘burst release effect.’ In the acidic tumor microenvironment (pH 5.5), nearly all the drug was released within 2 days. Furthermore, carriers modified with PEG2000 were internalized into HeLa cells via clathrin-mediated endocytosis, reducing lysosomal degradation. After loading dichloroacetic acid (DCA), the system demonstrated significant cytotoxicity at a concentration of 0.75 mg/mL, substantially enhancing therapeutic efficacy.

**Fig. 10:**
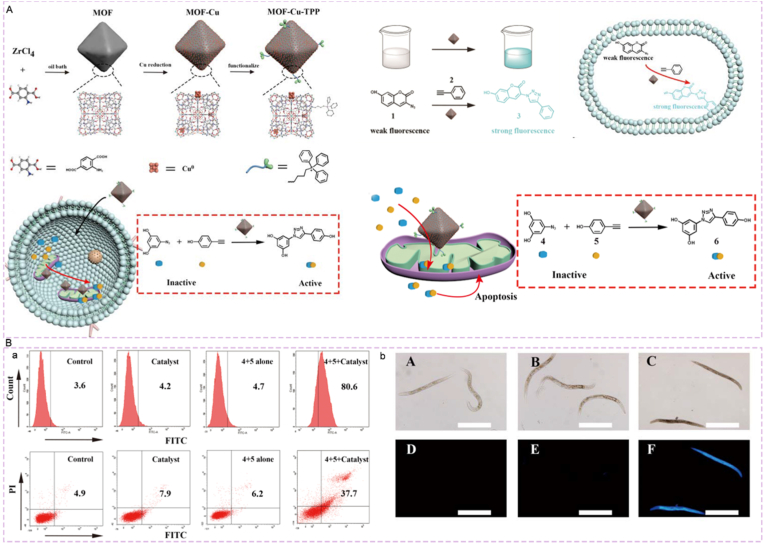
(A) Schematic diagrams of catalytic mechanisms and in-situ synthesis. (B) Bioactivity Validation of MOF-Cu-TPP (a) Cell-level experimental analysis. (b) Verification at the model organism level. Reprinted with permission from Ref. [180]. Copyright 2019 Wiley.

Qu et al. [180] constructed a mitochondria-targeting, in situ drug synthesis delivery system using CuAAC click chemistry. A Zr-based MOF was loaded with ultrafine copper nanoparticles to produce MOF-Cu, which was then modified with the mitochondrial targeting group triphenylphosphine (TPP) to yield the MOF-Cu-TPP catalyst (Fig. 10A). This system can target and accumulate in the mitochondria of MCF-7 cells, catalyzing the in situ synthesis of an active resveratrol derivative drug (6) from inert azide/alkyne precursors (4 and 5). It can induce mitochondrial damage and oxidative stress, significantly increasing the apoptosis rate. The precursors themselves are non-cytotoxic. The system exhibited good biocompatibility in *Caenorhabditis elegans* and mouse models (Fig. 10B). The in situ synthesized drug showed superior antitumor efficacy compared to direct drug administration, maximizing therapeutic effect while minimizing toxic side effects.

Giovanna Cutrone et al. [181] constructed a drug delivery system with an ‘invisible’ effect using CuAAC click chemistry. A dextran (DEX) backbone modified with propargyl carbamates (DEX-PC) was used. Through CuAAC reaction, azide-modified alendronate (ALN) and PEG were sequentially grafted, synthesizing a DEX-ALN-PEG comb-like copolymer. This copolymer was then non-covalently coated onto the surface of MIL-100 (Fe) nano MOFs via coordination between ALN and iron. This system could load DOX without affecting drug encapsulation and exhibited good stability in water and cell culture medium. It reduced human serum albumin (HSA) adsorption (to below 20 μg/mg) and significantly decreased macrophage uptake (only 24%-39% uptake within 4 h compared to the uncoated group), laying the foundation for enhanced drug delivery efficiency.

Wang et al. [182] constructed a cancer-cell-activated active targeted drug delivery system via CuAAC click reaction: Using Cu(II)-based MOF-199 as the carrier and Cu(I) catalyst precursor, they loaded two inert precursors (alkyne-modified TPA-alkyne-2+ and azide-modified MePy-N_3_), and prepared PMOF NPs by F-127 coating. Triggered by high concentrations of glutathione (GSH) in tumor cells, MOF-199 degrades to release the precursors, while Cu(II) is reduced to Cu(I) to catalyze the click reaction for synthesizing the mitochondria-targeting AIE photosensitizer TPATrzPy-3+. The synthesis yield of TPATrzPy-3+ reaches 70.5% in HeLa cells, which can efficiently generate ROS. Under light irradiation, it significantly increases the apoptosis rate of tumor cells. In the zebrafish tumor model, it achieves tumor-specific ablation with reduced phototoxicity, and the LC_50_ is 4 mM (much higher than 1 mM of direct administration).

##### 3.1.2.2 MSN

MSNs are characterized by ordered mesopores (pore size 2–10 nm), high chemical stability, and readily modifiable surface silanol groups, enabling the simultaneous encapsulation of hydrophobic drugs (e.g., paclitaxel) and hydrophilic nucleic acids [[183], [184], [185]].

**Fig. 11:**
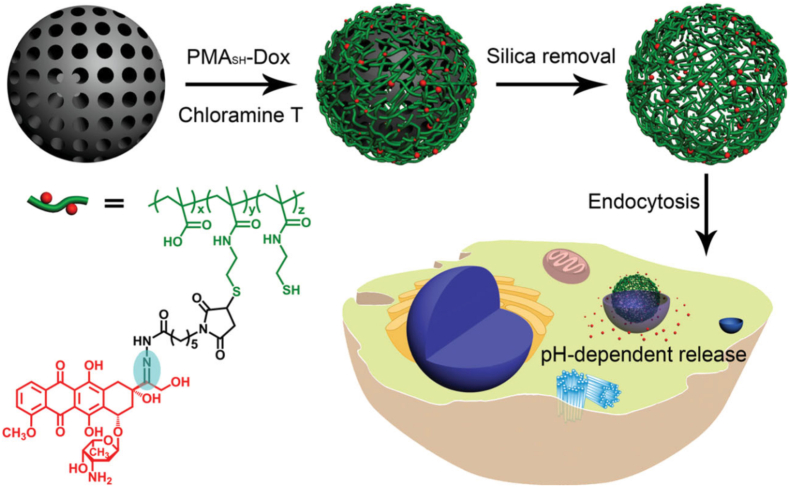
Schematic illustration of the modular assembly of drug-loaded polymer particles and pH-dependent drug release after endocytosis in a cancer cell. The structure of the polymer-drug conjugate is also shown. The labile hydrazone bond, which facilitates drug release, is shaded. Reprinted with permission from Ref. [186]. Copyright 2012 Wiley.

Cui et al. [186] constructed a pH-responsive active targeted drug delivery system via thiol-maleimide click reaction: Using thiolated poly(methacrylic acid) (PMA_sh_) as the carrier, they conjugated it with maleimide-modified doxorubicin (MAL-Dox) containing a pH-sensitive hydrazone bond through the click reaction to form PMA_sh_-Dox conjugates. The conjugates were infiltrated into mesoporous silica templates, followed by cross-linking of polymer chains and template removal to obtain drug-loaded particles (Fig. 11). The system is stable at physiological pH 7.2, with 80% drug release within 24 h at acidic pH 5.5. It has an IC_50_ of 28.5 nM against LIM1899 colorectal cancer cells (lower than 62.1 nM of free doxorubicin), can efficiently deliver drugs to cell nuclei and induce apoptosis, while the carrier itself shows no obvious cytotoxicity.

Xu et al. [187] constructed a self-fluorescent and stimuli-responsive drug delivery system via thiol-ene click reaction: Using large-pore mesoporous silica nanoparticles (LP) as the carrier, they anchored bis-acrylated curcumin as a "dual-function gating molecule" through the click reaction. After loading curcumin, the system was coated with F127 via self-assembly to obtain LPCC-C-F127. The system showed drug leakage below 20% at physiological pH 7.4 without GSH, and realized rapid drug release via β-thioester hydrolysis under tumor intracellular conditions (pH 5.5 and GSH). Curcumin served both as gating agent and fluorescent label; F127 coating enhanced the fluorescence intensity by 4.2 times and improved dispersibility. It reduced the viability of A549 cells to 48.8% at 200 μg/mL, achieving integration of imaging and therapy.

**Fig. 12:**
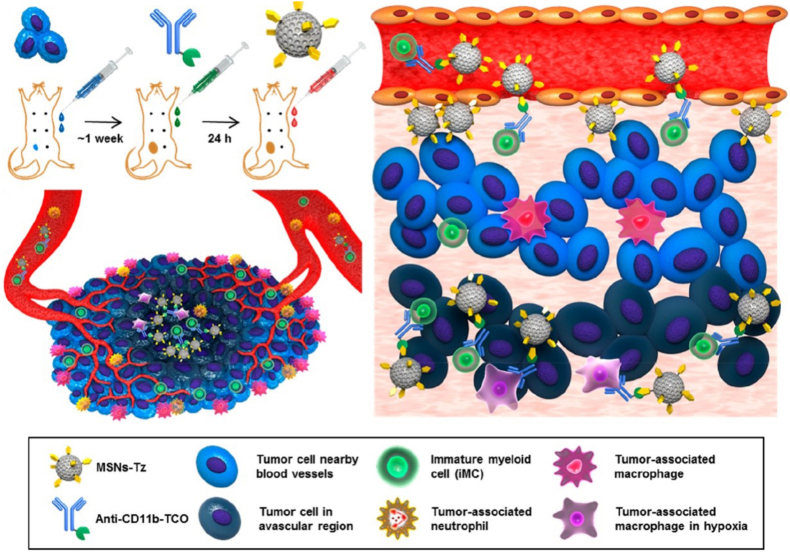
A schematic representation of CRAIT strategy used to enhance tumor penetration of drug-loaded NPs. Reprinted with permission from Ref. [188]. Copyright 2019 American Chemical Society.

Lee et al. [188] constructed an immune cell-mediated active targeted drug delivery system via IEDDA click reaction: TCO-modified anti-CD11b antibodies and tetrazine (Tz)-functionalized doxorubicin (DOX)-loaded mesoporous silica nanoparticles (MSNs-Tz) were intravenously injected sequentially, and MSNs-Tz were targeted to bind to the surface of CD11b^+^ myeloid cells through the click reaction (Fig. 12). The system delivers drugs to the avascular regions of tumors by virtue of the tumor-homing ability of CD11b^+^ cells, with its accumulation in avascular regions being 2-fold that of EPR effect-mediated delivery. In the 4T1 breast tumor model, it reduced tumor burden by approximately 50% without obvious hepatorenal function damage, showing significantly better therapeutic efficacy than free DOX and simple drug-loaded nanoparticles.

**Fig. 13:**
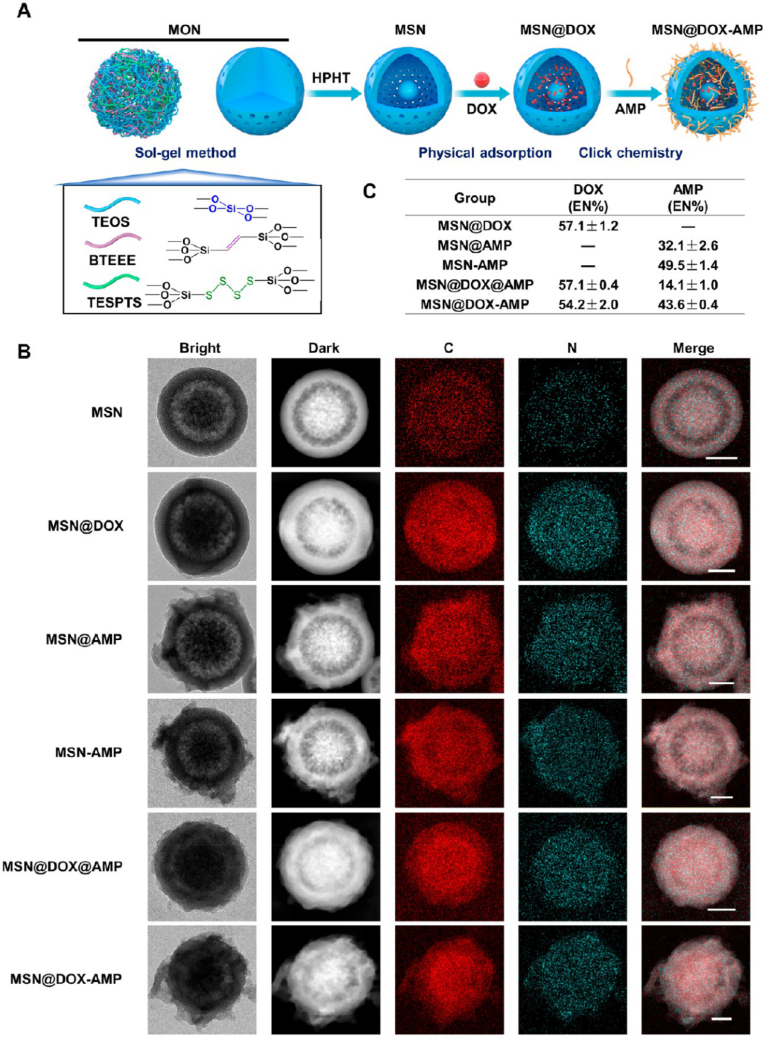
Preparation of the dual-drug-delivery mesoporous silica nanoparticles (MSNs). (A) Schematic illustration of the dual-drug-delivery MSNs. DOX was loaded by physical adsorption, and AMP was immobilized by thiol−ene click chemistry. (B) TEM images and EDS elemental mapping of the indicated drug delivery nanoparticles. The scale bar represents 100 nm. (C) Encapsulation ratios of DOX and AMP in the indicated nanoparticles. Reprinted with permission from Ref. [189]. Copyright 2023 American Chemical Society.

Ma et al. [189] constructed a GSH-responsive inhalable active targeted drug delivery system via thiol-ene click reaction (Fig. 13A): Using yolk-shell structured MSNs as the carrier (Fig. 13B), DOX was loaded by physical adsorption (Fig. 13C), and then thiol-modified antimicrobial peptide HHC36 was immobilized via thiol-ene click reaction to obtain MSN@DOX-AMP. After nebulized inhalation, the system can efficiently accumulate in the lungs, and rapidly release drugs under the action of high-concentration GSH in the tumor microenvironment. It achieves a 99.9% killing rate against intracellular and extracellular bacteria such as *Staphylococcus aureus*, and the inhibition rate against H1299 lung cancer cells is 13% higher than that of free DOX, while reversing the drug resistance of MCF-7/ADR cells. In the lung cancer-bacteria commensal mouse model, nebulized administration reduces the tumor volume by 95.4%, restores the alveolar area to 91.1% of the normal level, with no obvious hepatorenal toxicity.

#### 3.1.3 Bio-inspired carrier

Bioinspired nanodrug delivery systems, which mimic the structures, functions, or response mechanisms of biological entities, have significantly enhanced the precision and efficiency of drug delivery [190]. For example, biomimetic cell membrane-coated nanoparticles, by encapsulating natural cell membranes (e.g., erythrocyte membranes, tumor cell membranes, or bacterial membranes), provide carriers with immune evasion capabilities, prolonging their circulation time in vivo; and virus-like vectors, by mimicking the efficient invasion characteristics of viruses, are designed with nanostructures modified with targeting ligands, enhancing their recognition and internalization by specific cells. The application of click chemistry to Bio-inspired Carriers, such as exosomes or cell membrane-coated nanoparticles, necessitates ensuring biocompatibility [191]; for instance, when introducing azide groups via metabolic glycan engineering, the concentration of the precursor must be controlled to prevent cytotoxicity [192], and the click reaction must be designed to avoid disrupting the activity of membrane proteins or the immune evasion function of the carrier. In general, this review synthesizes the content of constructing active targeting drug delivery systems by conjugating targeting agents to bio-inspired carrier via click chemistry reactions, as shown in Table 4.

**Table 4:** Summary of click chemistry in Bio-inspired Carrier.

Type of DDSs	Click reaction	Deliver the load	Targeted area	Purpose	Ref
Viral-based	SPAAC	CpG adjuvant antigen peptideOVA257-280	Lung tumors	OVA257–280 is covalently bound to influenza Virus	196
	SPAAC	IRT	colorectal cancer	The covalent coupling of A-phage and D-IDNPs was realized	197
Cell-based	SPAAC	CAR T cells	Subcutaneous tumor	Conjugation of CAR T cells with immunomagnetic beads	199
	SPAAC	c(RGPBNPsDyK) peptide Curcumin	Ischemic brain injury area	Covalent attachment of c(RGDyK) to the exosome surface	200
	SPAAC	SILY peptides	Collagen area at the injury site	SILY peptide is covalently bound to the surface of stem cell-derived extracellular vesicles (EVs)	201
	SPAAC	Vascular-targeting peptide (DA7R) stem cell recruitment factor (SDF-1)	Areas of ischemic brain damage	Efficient attachment of DA7R peptide and SDF-1 factor to the surface of extracellular vesicles	202
Biomimetic	SPAAC	siRNA	Subcutaneous tumor	Attachment of RGD peptides to macrophage membranes	205
	SPAAC	BaTiO_3_ nanocubes	colorectal cancer	Modification of SAM@BTO nanoparticles onto the surface of VA cells	206
	Thiol-ene	Chondroitinase ABC Insulin-like Growth Factor 1	Damaged brain tissue	Fast and efficient construction of hydrogel networks	207

##### 3.1.3.1 Viral-based carrier

Virus-based nanodrug delivery systems achieve efficient and controllable delivery functions by engineering the natural infection mechanisms of viral capsids, coupled with click chemistry-mediated targeting modifications and precise drug loading strategies [193].After modification with acid-sensitive peptides or enzyme-responsive linkers, viral capsids can trigger drug release in the tumor microenvironment. Through genetic engineering, researchers can construct replication-defective attenuated viral vectors, such as by introducing premature termination codons (PTCs), and utilize click chemistry to precisely modify the viral surface with bioactive molecules such as antigen peptides and adjuvants, thereby achieving efficient antigen delivery, immune response activation, and modulation of immunosuppressive microenvironments [194,195].

**Fig. 14:**
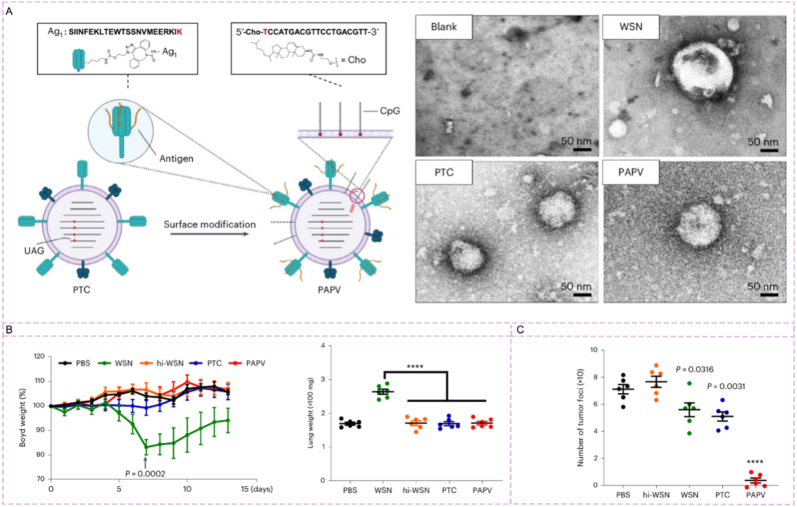
Generation and characterization of live but non-productive IAVs with chimeric antigenic peptides. (A) The construction principle of PAPV. (B) The left plot shows mouse body weight changes, the right plot detects cytokine levels in lung tissues. (C) Displays the number of lung tumor foci in the B16-F10 metastasis model across groups. Reprinted with permission from Ref. [196]. Copyright 2023 Springer Nature.

Ji et al. [196] constructed a lung-targeted cancer vaccine delivery system via SPAAC click reaction: Using an attenuated influenza virus (PTC virus) containing azide-bearing unnatural amino acid (NAEK) as the carrier, they covalently conjugated dibenzocyclooctyne (DBCO)-modified tumor antigen peptides (e.g., OVA_257-280_) to the virus surface through the click reaction, and then combined with cholesterol-modified CpG to obtain PAPV (Fig. 14A). After intranasal administration, the system accumulates in the lungs, with the antigen peptide uptake 200-fold higher than that of free peptides, the antigen presentation level of dendritic cells increased by 30-fold, and the number of antigen-specific CD8^+^T cells induced 30-fold that of the free peptide group. Compared with the wild-type influenza virus (WSN) group, the chimeric antigenic peptide influenza virus (PAPV) group showed no significant body weight loss in mice, with lung weight close to that of the control group (PBS) and no lung swelling (Fig. 14B). In the B16-F10 lung cancer metastasis model, it significantly reduces lung tumor foci; after combining with anti-PD-L1 nanobody, 10 out of 12 mice survive for more than 60 days without obvious lung toxicity (Fig. 14C).

Zheng et al. [197] constructed an intestinal microbiota-regulating active targeted drug delivery system via SPAAC click reaction: Using azide-modified *Fusobacterium nucleatum* (*F. nucleatum*)-specific phages (A-phages) as the targeting carrier, they covalently conjugated with DBCO-modified irinotecan (IRT)-loaded dextran nanoparticles (D-IDNPs) through the click reaction to form A-phage-D-IDNP complexes. After oral or intravenous administration, the system can target and bind to *F. nucleatum* in tumors for enrichment, with the intratumoral nanoparticle accumulation 3-fold higher than that of the non-targeted group. It can eliminate *F. nucleatum* (reducing the intratumoral colonization rate by 62.5%) and promote the proliferation of butyrate-producing bacteria (butyrate levels significantly increased). In the CT26 orthotopic colorectal cancer model, it achieves a tumor inhibition rate of 62%, extends the median survival of mice from 20 days to 42 days, and shows no obvious impact on the liver and kidney functions of piglets.

##### 3.1.3.2 Cell-based carrier

Cell-based nanodrug delivery systems construct biomimetic delivery vehicles by leveraging the biocompatibility and targeting functions of naturally occurring cellular vesicles, such as exosomes or cell membrane-derived vesicles [198]. The native membrane proteins of cellular vesicles (e.g., CD47) confer a “self-marker” ability that enables them to evade immune recognition, while the endogenous membrane structure can efficiently penetrate the vascular barrier and tumor matrix. Furthermore, the vesicle's microenvironment-responsive lipid layer can controllably release drugs at the tumor site via pH- or enzyme-triggered mechanisms, and the intercellular communication properties can be utilized to deliver therapeutic components to deep tumor cells or metastatic lesions. These systems integrate the low toxicity, high penetration, and intelligent drug release properties of cellular vesicles, providing a biomimetic solution for precise co-delivery in complex pathological environments.

**Fig. 15:**
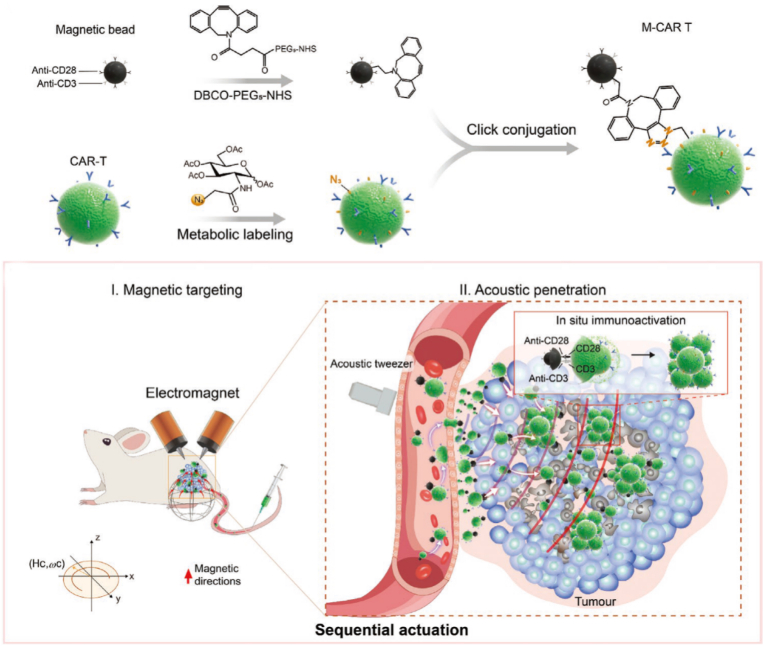
Schematic illustration of magnetic–acoustic sequentially actuated M-CAR Ts for programmable solid tumor targeting and enhanced immunotherapy. Reprinted with permission from Ref. [199]. Copyright 2023 Wiley.

Tang et al. [199] constructed a magneto-acoustic sequentially actuated CAR-T cell microrobot-based active targeted drug delivery system via SPAAC click reaction: CAR-T cells were metabolically labeled with azide groups (N_3_-CAR T), then covalently conjugated with DBCO-modified anti-CD3/CD28 immunomagnetic beads through click reaction to obtain M-CAR Ts (Fig. 15). After being targeted to the peritumoral region by magnetic guidance, the system was driven by acoustic tweezers to penetrate deep into tumors, resulting in a 6.6-fold increase in accumulated exogenous CD8^+^ CAR-T cells compared with the non-actuated group. The immunomagnetic beads could in situ activate CAR-T cell proliferation and cytokine secretion. In the CD19-SPCA1 subcutaneous tumor model, it reduced tumor volume by 80%, achieved 100% survival rate of mice at 32 days, and showed no obvious hepatorenal toxicity.

Tian et al. [200] constructed a targeted drug delivery system for cerebral ischemia via SPAAC click reaction: Using mesenchymal stem cell-derived exosomes as the carrier, DBCO groups were conjugated to the exosome surface via DBCO-sulfo-NHS, and then combined with azide-modified c(RGDyK) peptide through click reaction to obtain cRGD-Exo. After loading curcumin (cRGD-Exo-cur), the system was intravenously administered, which could target integrin αᵥβ_3_ in the ischemic brain region, with the ipsilateral/contralateral fluorescence intensity ratio reaching 19, nearly 2-fold higher than that of unmodified exosomes. It significantly inhibited the expression of pro-inflammatory factors such as TNF-α and IL-1β, reduced the fluorescence intensity of activated microglia, decreased cell apoptosis, and showed no obvious hepatotoxicity.

**Fig. 16:**
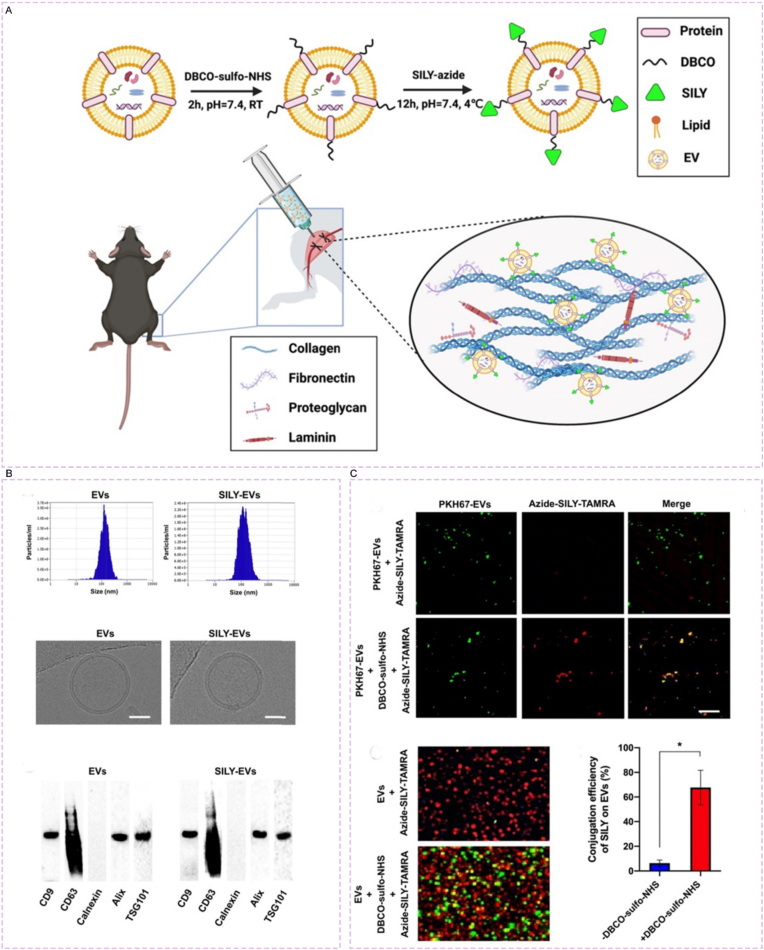
Preparation and characterization of SILY-EVs. (A) Schematic diagram of the study design. (B) Characterization of Physicochemical and Molecular Properties of EVs and SILY-EVs. (C) Verification of SILY-EVs Conjugation Effect. Reprinted with permission from Ref. [201]. Copyright 2022 Ivyspring.

Hao et al. [201] constructed a collagen-targeted extracellular vesicle (EV)-based active targeted drug delivery system via SPAAC click reaction: Using mesenchymal stem cell-derived EVs as the carrier, DBCO groups were conjugated to.

the EV surface via DBCO-sulfo-NHS, and then linked to azide-modified collagen-binding peptide SILY through click reaction to obtain SILY-Evs (Fig. 16A and B). After intramuscular injection, the system could target and bind to collagen in ischemic tissues (Fig. 16C). In the mouse hind limb ischemia model, its retention time was significantly prolonged (fluorescence signal still visible on day 7, while almost no signal in the unmodified EV group). It could promote M2 macrophage polarization, inhibit the expression of pro-inflammatory factors such as IFN-γ, increase the blood perfusion of ischemic sites by 40% and vascular volume by 35% compared with the unmodified EV group, accelerate muscle regeneration, and showed no obvious toxicity.

Ruan et al. [202] constructed a targeted drug delivery system for central nervous system injuries via SPAAC click reaction: Using extracellular vesicles (EVs) derived from M2 microglia as the carrier, DBCO groups were conjugated to the EVs surface via DBCO-PEG_4_-NHS, and then combined with azide-modified injured vascular targeting peptide DA7R and neural stem cell (NSCs)-recruiting factor SDF-1 through click reaction to obtain Dual-EV. After intravenous administration, the system could target vascular endothelial cells in the ischemic brain region, with the ipsilateral/contralateral fluorescence signal ratio significantly higher than that of unmodified EVs. It could recruit NSCs migration (migration rate 3.4-fold that of the control group) and induce their differentiation into neurons via miR-30b-3p et al. (Tuj1^+^ cells accounting for 9.4%). In the mouse ischemic stroke model, it reduced the cerebral infarct volume by approximately 79%, significantly decreased the neurological function score, and showed no obvious toxicity.

##### 3.1.3.3 Biomimetic carrier

Bioinspired nanodrug delivery systems represent an emerging biomedical technology that designs and constructs biocompatible, targeted, and functionalized nanocarriers by mimicking natural nanostructures or biological processes within living organisms, such as intercellular communication and immune evasion mechanisms [203,204]. These systems utilize biomaterials or biomolecules as building blocks to precisely deliver drugs to diseased sites while evading immune system recognition and clearance, thereby enhancing drug therapeutic efficacy and safety.

**Fig. 17:**
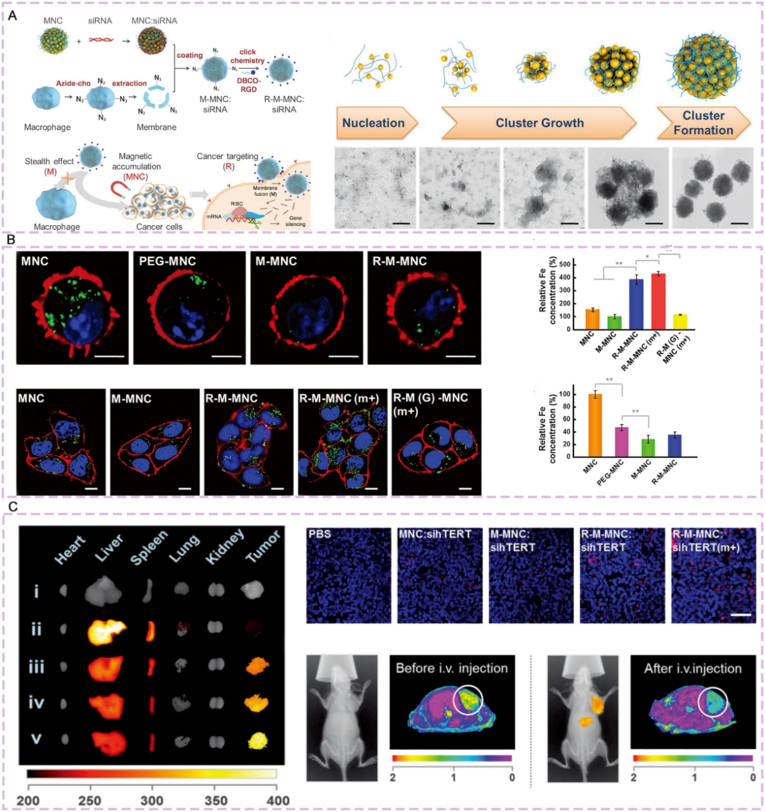
Characterization and performance of biomimetic magnetosome for siRNA delivery. (A) Schematic diagram of the construction principle and programmed delivery mechanism of MNC. (B) Characterization of MNC synthesis intermediates and verification of their effects at the cellular level. (C-) Verification of in vivo distribution and imaging application of MNC. Reprinted with permission from Ref. [205]. Copyright 2017 Wiley.

Zhang et al. [205] constructed a tumor-targeted biomimetic magnetosome drug delivery system via SPAAC click reaction: Using superparamagnetic Fe_3_O_4_ nanoclusters (MNCs) as the core to load siRNA, they coated azide-modified macrophage membranes via electrostatic interaction, and then conjugated DBCO-modified RGD peptides to the membrane surface through click reaction to obtain R-M-MNCs (Fig. 17A). After intravenous administration, the system could target tumor cell integrin αᵥβ_3_ with magnetic field assistance, showing significantly higher accumulation in tumors than unmodified carriers (Fig. 17B). It could efficiently silence the hTERT gene (mRNA expression inhibited to below 15%), greatly reduce tumor volume in MCF-7 tumor-bearing mouse models, significantly improve mouse survival rate, and cause no obvious hepatorenal function damage (Fig. 17C).

Fan et al. [206] constructed a targeted drug delivery system for colorectal cancer via SPAAC click reaction: Using *Veillonella atypica* (VA) as the carrier, after modification with azide-PEG_4_-NHS, it was conjugated with DBCO-modified *Staphylococcus aureus* membrane-coated barium titanate nanocubes (SAM@BTO) through click reaction to obtain VA-SAM@BTO. After oral administration, the system achieved dual targeting of colorectal cancer relying on the inflammatory targeting of SAM and hypoxic targeting of VA, with the fluorescence intensity at the tumor site 2.87-fold higher than that of the SAM@BTO group. Under ultrasonic stimulation, BTO catalyzed multiple reactions to generate ROS and CO, which synergized with VA to metabolize lactic acid, achieving a tumor inhibition rate of 90.36%. The proportion of M1-type macrophages increased from 11.4% to 28.5%, the proportion of CD8^+^ T cells significantly increased, and there was no obvious hepatorenal toxicity.

**Fig. 18:**
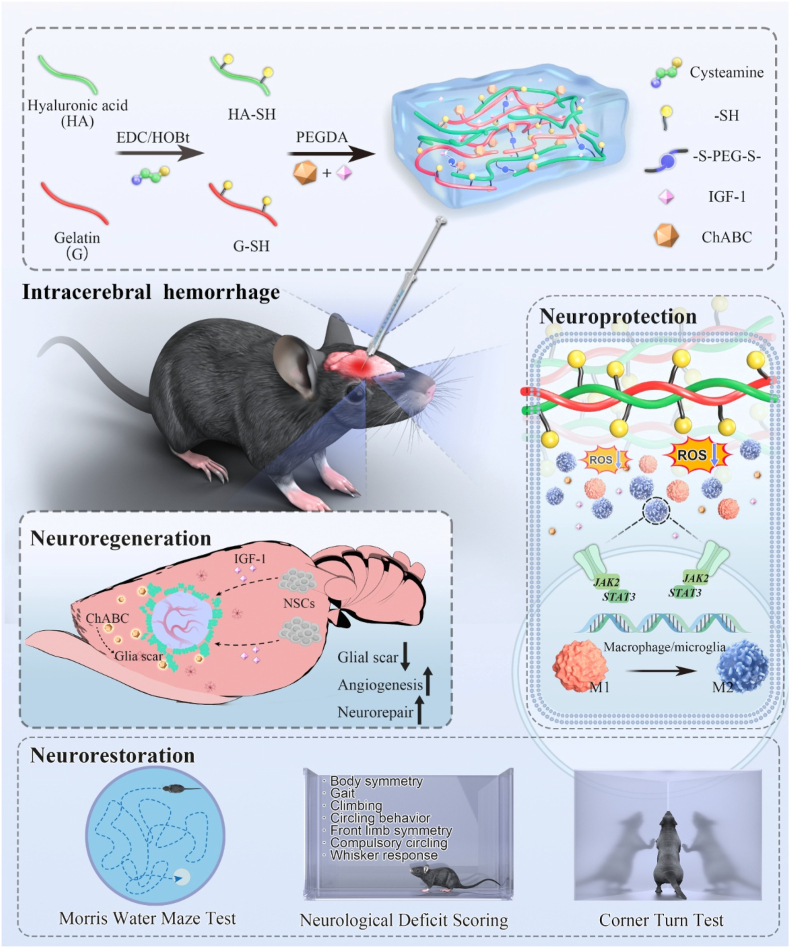
Outline a combined strategy of brain neuroprotection and endogenous neuroregeneration for enhanced intracerebral hemorrhage treatment. Reprinted with permission from Ref. [207]. Copyright 2024 Elsevier.

Xu et al. [207] constructed a targeted drug delivery system for intracerebral hemorrhage via thiol-ene click reaction (Fig. 18): Using thiolated gelatin (G-SH) and thiolated hyaluronan (HA-SH) as raw materials, they cross-linked with polyethylene glycol diacrylate (PEGDA) through the click reaction to form a biomimetic hydrogel (GEL), which was loaded with chondroitinase ABC (ChABC) and insulin-like growth factor 1 (IGF-1). After injection, the system could target intracerebral hemorrhage lesions, efficiently scavenge reactive oxygen species via thiol groups (ROS scavenging rate over 97%), and regulate macrophage M2 polarization through the JAK2/STAT3 pathway. It could reduce glial scar formation (GFAP-positive area decreased by about 44% compared with the control group), promote the proliferation and migration of endogenous neural stem cells. In the mouse intracerebral hemorrhage model, it significantly improved the repair efficiency of neurons and myelin, enhanced cognitive and motor functions, and showed no obvious blood toxicity.

### 3.2 In vivo strategy: in vivo dynamic targeting based on click chemistry

**Scheme 2:**
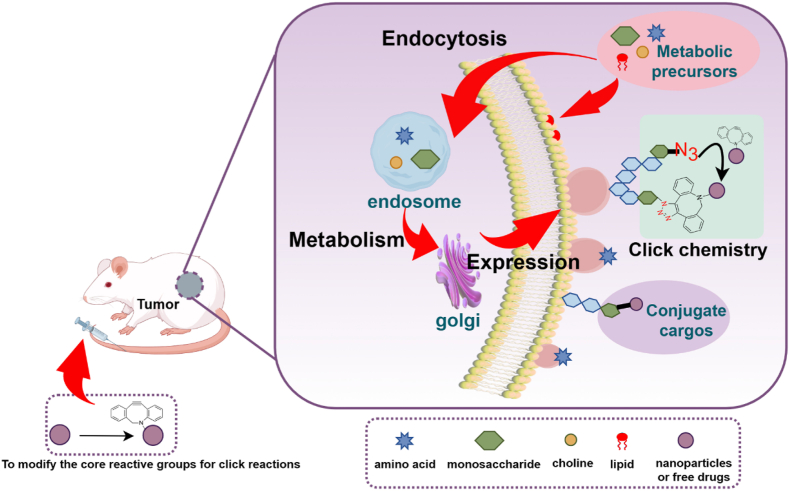
Illustration of the in vivo dynamic targeting based on click chemistry. First, metabolic precursors are internalized by cells. Second, the precursors participate in cellular metabolic processes. Third, bioorthogonal groups are displayed on the cell surface. Finally, cargo molecules are conjugated to cells via click reactions.

The in vivo strategy relies on the synergistic action of metabolic engineering and click chemistry to construct an in vivo dynamic active targeting drug delivery system, primarily focusing on the precise sequential achievement of ‘target labeling’ and ‘drug anchoring.’ In specific applications, this strategy is implemented in two sequential steps. The first step is the metabolic labeling phase: inert bioorthogonal click chemistry groups (e.g., azide N_3_) are introduced into the body through local injection or systemic delivery mediated by nanocarriers. These precursors are then taken up by target cells (such as cancer cells, bacteria, or immune cells) and integrated into their cell membrane structures, including glycoproteins, phospholipids, or glycans, via inherent cellular metabolic pathways, such as the sialic acid biosynthesis pathway or phospholipid synthesis pathway. Unnatural sugars bind to cell surface glycans through the glucose metabolic pathway. Lipid precursors are embedded in cell membranes through phospholipid synthesis pathways or lipid insertion. Functionalized amino acids bind to membrane proteins through protein synthesis pathways. This results in target cell surfaces efficiently and specifically displaying bioorthogonal groups without interfering with normal physiological functions. The second step is the click conjugation phase: after metabolic labeling is complete, drug-loaded nanocarrier systems modified with complementary inert click chemistry groups (e.g., dibenzocyclooctyne DBCO, trans-cyclooctene TCO, tetrazine Tz) are administered intravenously. Through copper-free click reactions (e.g., SPAAC, iEDDA), the drug-loaded systems form efficient and specific covalent bonds with the bioorthogonal groups on the target cell surfaces, achieving drug enrichment and retention at the target siteas, shown in Scheme 2. This strategy can enhance labeling selectivity by optimizing the delivery method of metabolic precursors and improve drug targeting efficiency by leveraging the high reaction rate of click chemistry [208,209]. It effectively addresses issues in traditional delivery systems, such as poor tumor penetration and off-target distribution, providing core technological support for precision cancer therapy, bacterial infection intervention, and immune modulation. In general, this review synthesizes the in vivo dynamic targeting DDSs based on click chemistry, as shown in Table 5.

**Table 5:** Summary of click chemistry-targeted DDSs.

Metabolic Precursor	Group	Click reaction	Deliver the load	Targeted area	Purpose	Ref
DSPE-PEG-N_3_	N_3_	SPAAC	OVA257-264 peptide poly(I:C) adjuvant	Lymph node microenvironment	Achieving active lymph node targeting	187
AC_3_ManNAz	N_3_	SPAAC	Ferroptosis inducer RSL3 DHA	Subcutaneous tumor	Toward specific labeling of tumor cells	188
Ac_4_ManNTz	Tz	IEDDA	Dox PROTACs(ARV771)	Subcutaneous tumor	Achieve the specific "click-release" of TCO-modified prodrugs on the surface of tumor cells to activate drug activity	189
Ac_3_ManNAz-BO	N_3_	SPAAC	Necroptosis activator C6 NO	The surface of TCSCs	Enable the specific coupling of DLip@NO/C6 with the artificial azide receptors introduced by glycoengineering on the surface of TCSCs	190
Ac_4_ManNAz	N_3_	SPAAC	Antimicrobial peptide LL37	Inflammatory sites caused by systemic infection	Enable precise coupling of DBCO-LL37 with M24N_3_ cells at the inflammatory site	191

**Fig. 19:**
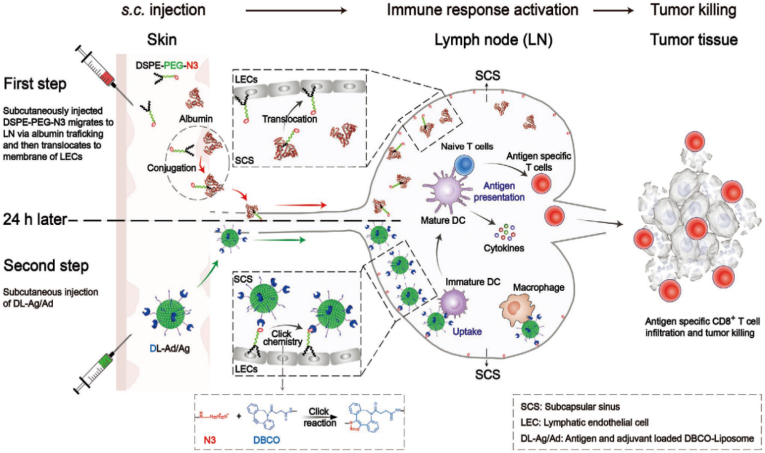
Schematic illustration of the active lymph-node accumulation cancer vaccine system based on click chemistry. Reprinted with permission from Ref. [210]. Copyright 2021 Wiley.

Qin et al. [210] developed an active targeting drug delivery system based on a two-step strategy via SPAAC reaction: First, DSPE–PEG–N3 was injected subcutaneously, which migrated to lymph nodes via the albumin RH mechanism and displayed azide groups on the surface of lymphatic endothelial cells (LECs); 24 h later, liposomes modified with DBCO and loaded with OVA_257-264_ peptide and poly(I:C) were injected, and the liposomes bound to the azide groups on LECs via SPAAC reaction to achieve lymph node accumulation (Fig. 19). This system enhanced the uptake rate of the vaccine by antigen-presenting cells, induced a stronger CD8^+^ T cell response, completely inhibited lung metastasis in a melanoma model, and increased the 60-day survival rate of mice by 100% compared with the group without azide targeting.

**Fig. 20:**
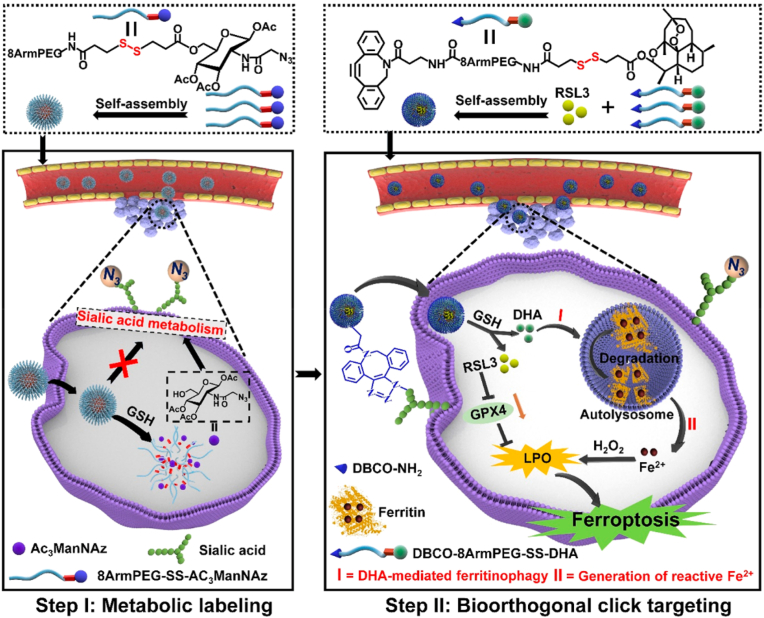
Schematic illustration for the molecular structures of 8ArmPEG-SS-AC3ManNAz and DBCO-8ArmPEG-SS-DHA@RSL3 nanoassemblies and therapeutic mechanisms. Reprinted with permission from Ref. [211]. Copyright 2022 American Chemical Society.

Li et al. [211] developed an active targeting drug delivery system based on the SPAAC reaction: First, 8ArmPEG-SS-AC_3_ManNAz nanoassemblies were intravenously injected, which released AC_3_ManNAz under the action of high-concentration GSH in tumor cells, enabling tumor cells to express azide groups on their surface via metabolic glycoengineering; subsequently, DBCO-8ArmPEG-SS-DHA@RSL3 nanoassemblies loaded with ferroptosis inducer RSL3 and ferritinophagy initiator DHA were injected, and targeted delivery was achieved by binding to azide groups on the tumor cell surface via the SPAAC reaction (Fig. 20). In the 4T1 tumor model, this system reduced the final tumor volume to only about 140 mm^3^, prolonged the median survival of mice by 15 days compared with the PBS group, and showed no obvious systemic toxicity.

Chen et al. [212] developed an active targeting drug delivery system based on the iEDDA reaction: First, tetraacetylated tetrazine mannosamine (Ac_4_ManNTz) was efficiently modified onto the surface of cancer cells via metabolic glycoengineering to construct tetrazine (Tz)-functionalized artificial receptors; subsequently, Tz-labeled cancer cells specifically bound to TCO-caged prodrugs (e.g., DOX, ARV771) through the iEDDA reaction, enabling local activation of prodrugs and release of active drugs. This system enhanced the selectivity of TCO-doxorubicin by 10-fold compared to unlabeled cells, and could degrade approximately 80% of BRD4 protein at a TCO-ARV771 concentration of 100 nmol/L, significantly improving the precision and safety of tumor-targeted therapy.

**Fig. 21:**
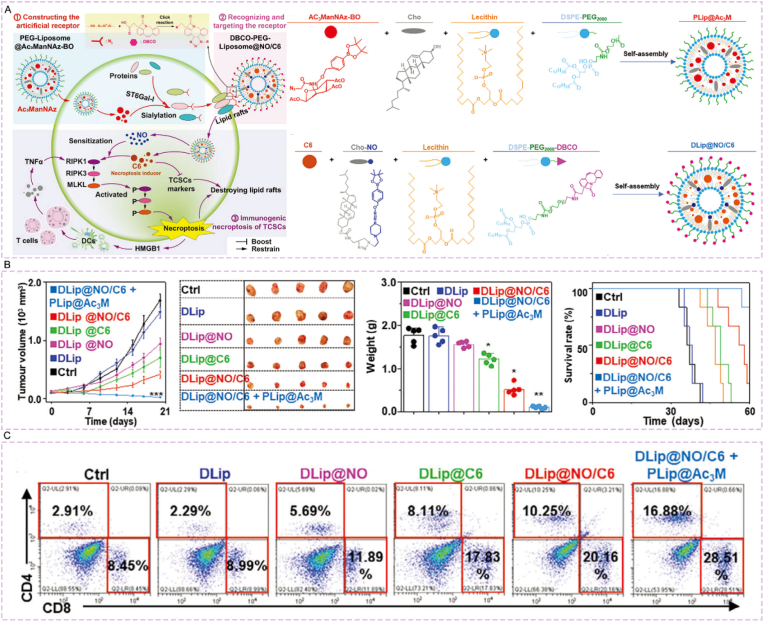
(A) Schematic diagram of the mechanism of action and nanoplatform design. (B) Tumor Growth and Survival Curves. (C) Statistical chart of T cell infiltration ratio. Reprinted with permission from Ref. [213]. Copyright 2024 Wiley.

Li et al. [213] developed an active targeting drug delivery system based on the strain-promoted SPAAC reaction: First, triacetylated N-azidoacetyl-D-mannosamine with ROS-responsive boronic ester group (Ac_3_ManNAz-BO) was loaded into PEGylated liposomes (PLip@Ac_3_M), and azide artificial receptors were expressed on the surface of triple-negative breast cancer stem cells (TCSCs) via metabolic glycoengineering; subsequently, liposomes modified with DBCOand loaded with necroptosis activator C6 and NO prodrug (DLip@NO/C6) were injected, which targeted and bound to TCSCs via the SPAAC reaction (Fig. 21A). This system reduced the final tumor volume to only about 22.9 mm^3^ (Fig. 21B), increased the tumor infiltration rate of CD8^+^ T cells by 16.88% (Fig. 21C), effectively inhibited tumor metastasis and prolonged the survival time of mice.

## 4 Summary and outlook

Click chemistry demonstrates multidimensional application value in the construction of active targeting drug delivery systems, primarily achieving precise targeting and efficient delivery through diverse reaction types and strategies. Commonly used reactions include CuAAC, SPAAC, and IEDDA. Among these, CuAAC achieves efficient conjugation of biomolecules with rapid kinetics, SPAAC avoids copper toxicity and is suitable for in vivo applications, and IEDDA, due to its ultra-fast reaction rate, serves as a core for dynamic in vivo targeting. At the strategic level, the in vitro strategy involves modifying the surface of carriers with groups such as N_3_ and efficiently coupling them with complementary groups (DBCO, TCO, etc.) conjugated to targeting ligands or drug precursors. The in vivo strategy combines metabolic engineering with copper-free click reactions, wherein bioorthogonal group precursors are first labeled onto the target cell surface via metabolic precursors, followed by the enrichment of drug-loaded systems at the target site through SPAAC or IEDDA reactions.

The most fundamental challenge for the U.S. Food and Drug Administration (FDA) in approving click chemistry-based therapeutics lies in the stringent validation required for the in vivo specificity of the click reaction, the metabolic safety of any byproducts, and the spatio-temporal coordination of dual-component drug systems. This rigorous validation is necessary to ensure highly efficient drug activation specifically within the tumor microenvironment while simultaneously precluding potential toxicities induced by non-specific reactions in normal tissues. Currently, click chemistry applications in FDA clinical trials have progressed from the “proof-of-concept” phase to the “efficacy validation” stage. SQ 3370 represents the first click chemistry anticancer drug to enter human clinical trials [214]. Designed based on IEDDA reaction, it comprises a tumor-site-injected Tz biopolymer and a systemically administered TCO doxorubicin prodrug. The active drug is released at the tumor site via the click reaction. Phase I/IIa studies have demonstrated that SQ 3370 is safe up to 12 times the conventional dose of doxorubicin. TGW101 is the first FDA-IND approved click-release ADC [215], developed using bioorthogonal click chemistry to target the non-internalizing antigen TAG-72. This system releases the cytotoxic payload MMAE via a click reaction between the ADC and a small-molecule trigger. Phase I trials are currently recruiting patients with advanced solid tumors to evaluate safety and maximum tolerated dose, successfully overcoming the limitations associated with the target internalization dependence of traditional ADCs.

Click chemistry, while demonstrating advantages such as high specificity and low toxicity in constructing actively targeted cancer therapies, still faces limitations. Firstly, the expression density and stability of chemical receptors introduced via metabolic glycan engineering within tumors are constrained by the in vivo metabolic rate of the precursor drug and fluctuations in enzyme activity, leading to unpredictable target abundance [216]. Secondly, off-target reactions of click ligands in circulation and non-specific adsorption driven by hydrophobicity result in elevated background signals in normal tissues, and there is currently no mechanism to instantaneously “switch off” the reaction in vivo [217]. Finally, the temporal and spatial matching between click reaction kinetics and drug release dynamics is inadequate, causing significant fluctuations in the intratumoral activation half-life, which hinders reproducible dosing [218].

Of particular note are next-generation click chemistry technologies. SeNEx, utilizing selenium (Se) as a linkage center, achieves highly efficient C-Se bond construction via a selenium-nitrogen exchange reaction, offering advantages such as modularity, mild reaction conditions, excellent functional group compatibility, and suitability for nanomolar-scale synthesis and bioconjugation. SuFEx, on the other hand, benefits from metal-free participation, the coexistence of high stability and reactivity in the S-F bond, and the ability to react directly with naturally occurring functional groups in biomolecules. Both platforms are applicable in scenarios such as the modification of tumor-targeting nanomaterials and prodrug activation, holding the potential to overcome limitations of existing click chemistries, such as immunogenicity and biological instability. Future advancements could involve developing tumor microenvironment-responsive smart metabolic precursors to enhance labeling specificity, designing novel click chemistry pairs with tunable reaction rates to match in vivo drug delivery kinetics, and combining these with the precise delivery capabilities of nanocarriers to achieve spatio-temporal control over click reactions. Furthermore, the convergence of click chemistry and artificial intelligence holds significant promise, as machine learning algorithms can predict optimal reaction conditions and ligand configurations, potentially accelerating the development of nanocarriers.

## CRediT authorship contribution statement

**Yonghui Liu:** Conceptualization, Project administration, Validation, Visualization, Writing – original draft. **Wei Zhang:** Visualization. **Yanan Wu:** Software. **Dong Wan:** Writing – review & editing. **Jie Pan:** Funding acquisition, Writing – review & editing.

## Declaration of competing interest

The authors declare the following financial interests/personal relationships which may be considered as potential competing interests:Jie Pan reports was provided by Tiangong University. Jie Pan reports a relationship with Tiangong University that includes:. If there are other authors, they declare that they have no known competing financial interests or personal relationships that could have appeared to influence the work reported in this paper.

## Data Availability

No data was used for the research described in the article.
